# Prediction of genetic relatedness of *Escherichia coli* using neighbor typing: a tool for rapid outbreak detection

**DOI:** 10.1128/aac.01071-25

**Published:** 2026-01-26

**Authors:** Amanda C. Carroll, Leanne Mortimer, Hiren Ghosh, Sandra Reuter, Hajo Grundmann, Karel Brinda, William P. Hanage, Angel Li, Aimee Paterson, Andrew Purssell, Ashley M. Rooney, Noelle R. Yee, Bryan Coburn, Shola Able-Thomas, Martin Antonio, Allison McGeer, Derek R. MacFadden

**Affiliations:** 1 The Ottawa Hospital Research Institute 10055, Ottawa, Ontario, Canada; 2 Children’s Hospital of Eastern Ontario Research Institute, Ottawa, Ontario, Canada; 3 University of Ottawa 6363, Ottawa, Ontario, Canada; 4 Medical Center – University of Freiburg 9174, Freiburg, Germany; 5 Inria, Irisa, Univ. Rennes, Rennes, France; 6 Harvard T.H Chan School of Public Health, Harvard University 1812, Cambridge, Massachusetts, USA; 7 Sinai Health System 518775, Toronto, Ontario, Canada; 8 The Ottawa Hospital, Ottawa, Ontario, Canada; 9 Institute of Medical Microbiology, University of Zurich 27217, Zurich, Switzerland; 10 University Health Network 7989, Toronto, Ontario, Canada; 11 The University of Toronto, Toronto, Ontario, Canada; 12 MRC Unit The Gambia at the London School of Hygiene and Tropical Medicine 47969 , Banjul, Gambia; 13 Centre for Epidemic Preparedness and Response, London School of Hygiene & Tropical Medicine 4906, London, United Kingdom; 14 Department of Infection Biology, Faculty of Infectious and Tropical Diseases, London School of Hygiene & Tropical Medicine 218289 , London, United Kingdom; University of Pennsylvania Perelman School of Medicine, Philadelphia, Pennsylvania, USA

**Keywords:** genetic relatedness, outbreak detection, rapid diagnostics, metagenomics, nanopore, genomics

## Abstract

Identifying the genetic relatedness of resistant bacterial pathogens in healthcare settings can help identify undetected transmission events and outbreaks. However, current methods are time- and resource-intensive. We evaluated a rapid neighbor typing method paired with long-read sequencing for assessment of genetic relatedness. Utilizing a data set of primary clinical samples and published isolate data from two outbreaks of *Escherichia coli*, we applied genomic neighbor typing of long-read sequence data to rapidly estimate genetic relatedness. We assessed the correlation between neighbor typing predicted genetic distance and pairwise genetic distance from short-read draft whole genomes for all sample pairs. Predicted genetic trees using neighbor typing were compared to reference genetic trees generated using mash distances and maximum-likelihood (ML) methods to assess the extent of agreement, along with metrics of cluster similarity (cluster comparability and Baker’s gamma index [BGI]) and tree topology similarity (generalized Robinson-Foulds [GRF] metric). For all three data sets, we found strong correlations between the reference methods and predicted genetic distances (Spearman’s rho = 0.75–0.95, *P* < 0.001), which improved when using a lineage score-informed approach (Spearman’s rho = 0.93–0.94, *P* < 0.001). Predicted genetic trees and clusters from neighbor typing were comparable to those generated using either *mashtree* or an ML method, with a range of cluster comparability of 85.8–99.5%, BGIs of 0.8–0.95, and GRF values of 0.34–0.8. Pairing the neighbor typing method with long-read sequencing can enable accurate predictions of the relatedness of *E. coli* samples and isolates, and could potentially be used as a rapid outbreak surveillance tool.

## INTRODUCTION

Antibiotic-resistant organisms (AROs), and the infections they cause, pose a growing health threat. Antimicrobial resistance (AMR) in bacteria threatens both community-dwelling and hospitalized populations and can transmit asymptomatically in both settings (1, 2). Approximately 8% of inpatients experience one or more hospital-acquired infection(s) (3). To detect potential routes of transmission and guide infection control strategies, there is a need to determine how closely pathogens are related (4), yet current microbiologic approaches to ascertaining relatedness are often slow and/or resource-intensive (5). Genomic surveillance has emerged as an attractive approach, but many methods still rely on short-read sequencing, which is often too slow and costly to be practically useful for a real-time surveillance program. However, determining the relatedness of isolates by combining rapid, long-read sequencing with *k-mer*-based prediction algorithms is an alternative approach that could overcome these limitations (6).

Whole-genome sequencing (WGS) with phylogenetic analysis is the current reference standard for identifying transmission events (7), but there remain some key challenges for its routine use. Typical short-read WGS requires specialized infrastructure within hospitals that may not be available or feasible to maintain and operate (7). Even when available, the financial cost for WGS, including bioinformatic analysis and interpretation, can be cost-prohibitive for routine use. Most importantly, typical short-read WGS workflows are time-intensive, meaning that there will be a delay in obtaining and interpreting the results (8, 9). Sequencing *k-mer-*based analysis is a rapid, more computationally efficient, and logistically simpler approach that has the potential to reduce some barriers to implementation in hospital laboratories (10, 11). Previously, one such *k*-mer-based approach has been used for “neighbor typing” (12). This software uses *k*-mer databases of resistance-associated sequence elements (RASE) to predict an unknown sample’s best matching lineage or “neighbor” in order to predict antibiotic susceptibility phenotype in key pathogens through association between relatedness and phenotype (12, 13). We have previously demonstrated the use of neighbor typing paired with rapid long-read sequencing using Nanopore in order to predict antibiotic susceptibility phenotypes in *Streptococcus pneumoniae*, *Escherichia coli*, *Neisseria gonorrhoeae,* and *Klebsiella* spp. (12, 13). As neighbor typing can identify a closely related lineage for unknown samples, it could potentially be used to identify outbreaks, as any samples drawn from the same short transmission chain must be by definition closely related to each other, and so will match to the same neighbor in a database representing the genomic diversity of the species. We found in our previous work that the typical time for sample preparation and sequencing to obtain adequate reads to make informed conclusions was approximately 6 h, which demonstrates the quick turnaround time in which results can be obtained and acted upon, which is necessary for rapid diagnostic and surveillance tools (13). Such tools fall within a burgeoning field of pan-genomic epidemiology, where the relatedness and transmission dynamics of species with highly dynamic genomes and measured over shorter time scales can be improved by accounting for both the core and accessory genomes (14). Since mutations may not arise rapidly enough to be assessed using core genome single-nucleotide polymorphisms, transmission in limited areas over minimal timescales may benefit by using the additional information found in the accessory genome. This highlights the need to use tools that can incorporate data from all aspects of the genome.

In this retrospective study, we demonstrate the use of *k*-mer-based neighbor typing of long-read sequence data to predict relatedness between primary specimens and isolates using three *E. coli* data sets from both non-outbreak and outbreak settings.

## RESULTS

### Correlation between predicted genetic distance and reference standard genetic distance

**Fig 1:**
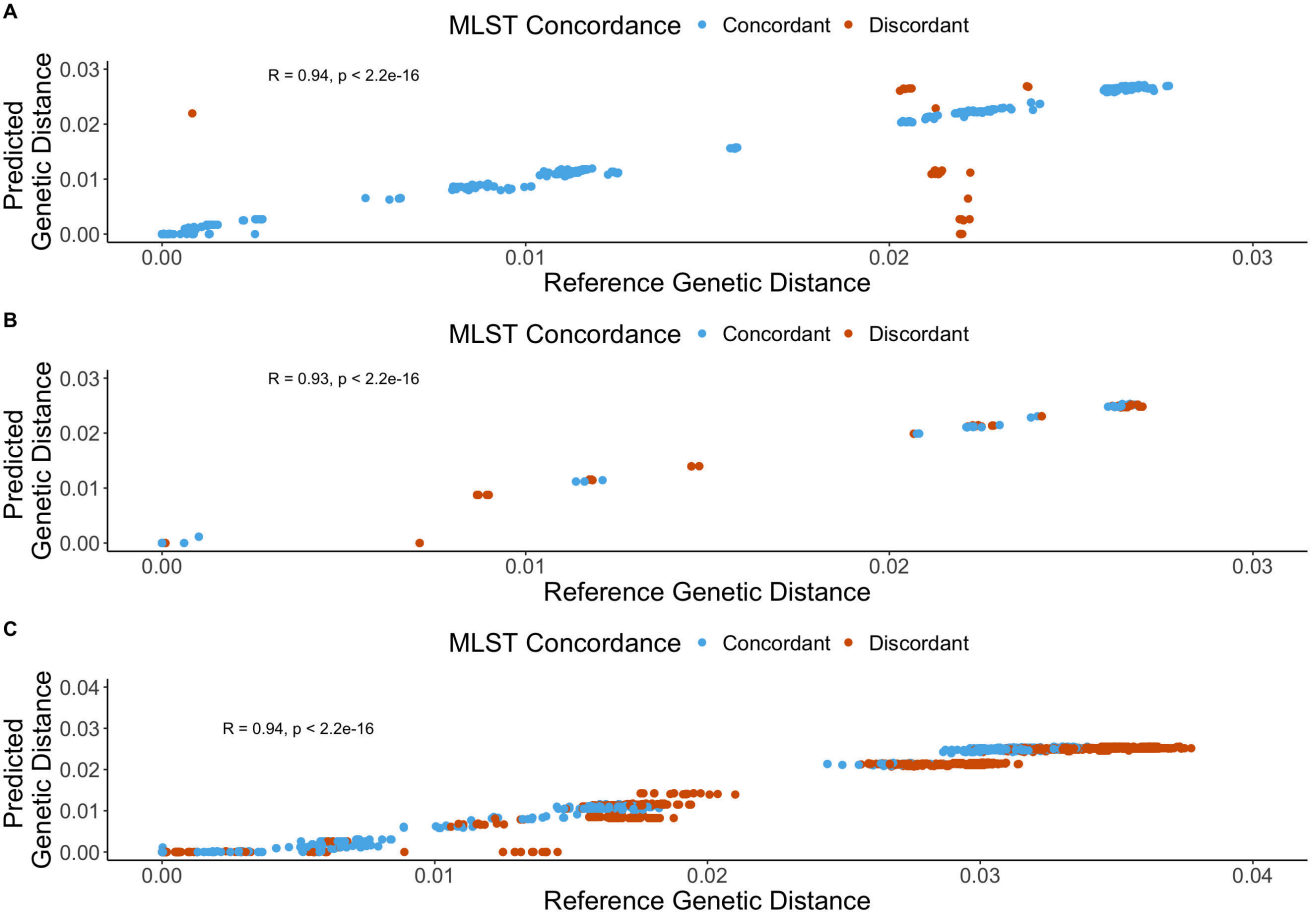
Plot of predicted and reference standard genetic distances for *Escherichia coli* using a lineage score (LS)-informed approach (LS ≥ 0.5). Panel (**A**) represents the *surveillance* data set, panel (**B**) represents the *short* outbreak data set, and panel (**C**) represents the *long* outbreak data set. Blue data points are predictions for concordant calls, and orange data points are predictions based on discordant calls.

We first evaluated the correlation between the predicted genetic distances derived using the neighbor typing method and the reference standard pairwise genetic distance for all samples. Using a lineage score (LS)-informed approach, we found strong correlations across all data sets, each with a Spearman’s rho of 0.93 (95% confidence interval [CI]: 0.91–0.95) (*short* outbreak data set) or 0.94 (both *surveillance* and *long* outbreak data sets) (*surveillance* 95% CI: 0.93–0.95; *long* 95% CI: 0.93–0.94) (Fig. 1). Without LS stratification, we also found that for all data sets there were strong correlations between neighbor typing predicted genetic distances and reference method genetic distance, with a Spearman’s rho of 0.81 (95% CI: 0.72–0.83) for the *surveillance* data set, 0.75 (95% CI: 0.72–0.78) for the *short* outbreak data set, and 0.95 (95% CI: 0.95–0.95) for the *long* outbreak data set (Fig. S2). Stratification by using only the clinical samples/isolates where the neighbor typing-predicted multi-locus sequencing type (MLST) was concordant with the sequenced isolate MLST for both pairs showed near-perfect correlation compared to the reference method across the three data sets, with a Spearman’s rho of 0.99 (95% CI: 0.98–0.99) (*surveillance* data set), 0.99 (95% CI: 0.98–0.99) (*short* outbreak data set), and 0.92 (95% CI: 0.91–0.93) (*long* outbreak data set) (Fig. S3). We also plotted the same data using single-nucleotide polymorphism (SNP) distances rather than genetic distances (Fig. S4 to S6). We further assessed the histograms of the reference genetic distances for each data set in order to describe the distributions of distances (Fig. S6).

### Concordance of clustering between genetic trees relative to the phylogenetic tree

**Fig 2:**
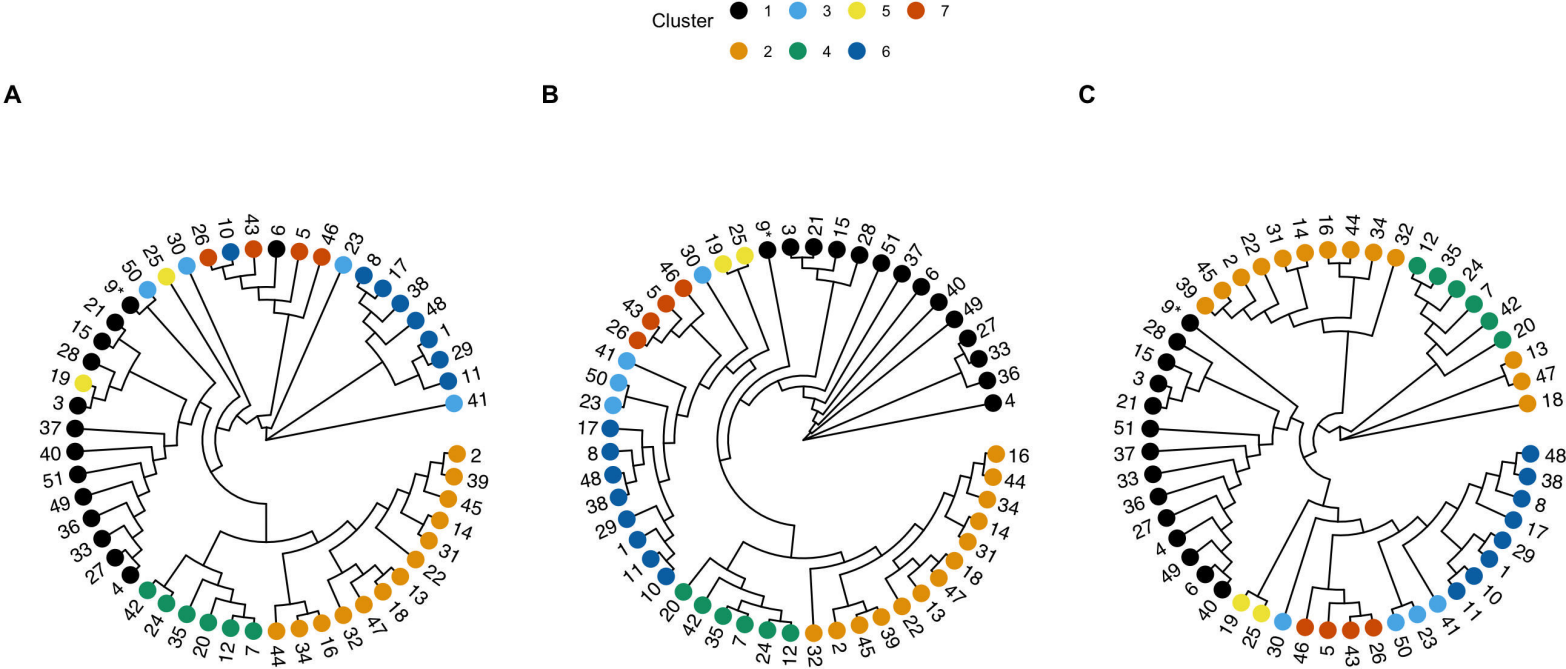
Circular genetic trees assembled for the *surveillance* data set, including (**A**) neighbor typing predicted genetic tree using neighbor typing; (**B**) reference genetic tree created using *mashtree*; and (**C**) reference maximum-likelihood (ML) phylogeny created using *PanACoTA*. Tips are colored by cluster, as determined using *rhierBAPS*, and the clusters from the ML reference method are mapped onto the best match trees for comparison. A consistent sample number is labeled at the tips for ease of comparison of sample locations between trees. Asterisk (*) used to denote sample 9 from sample 6.

**Fig 3:**
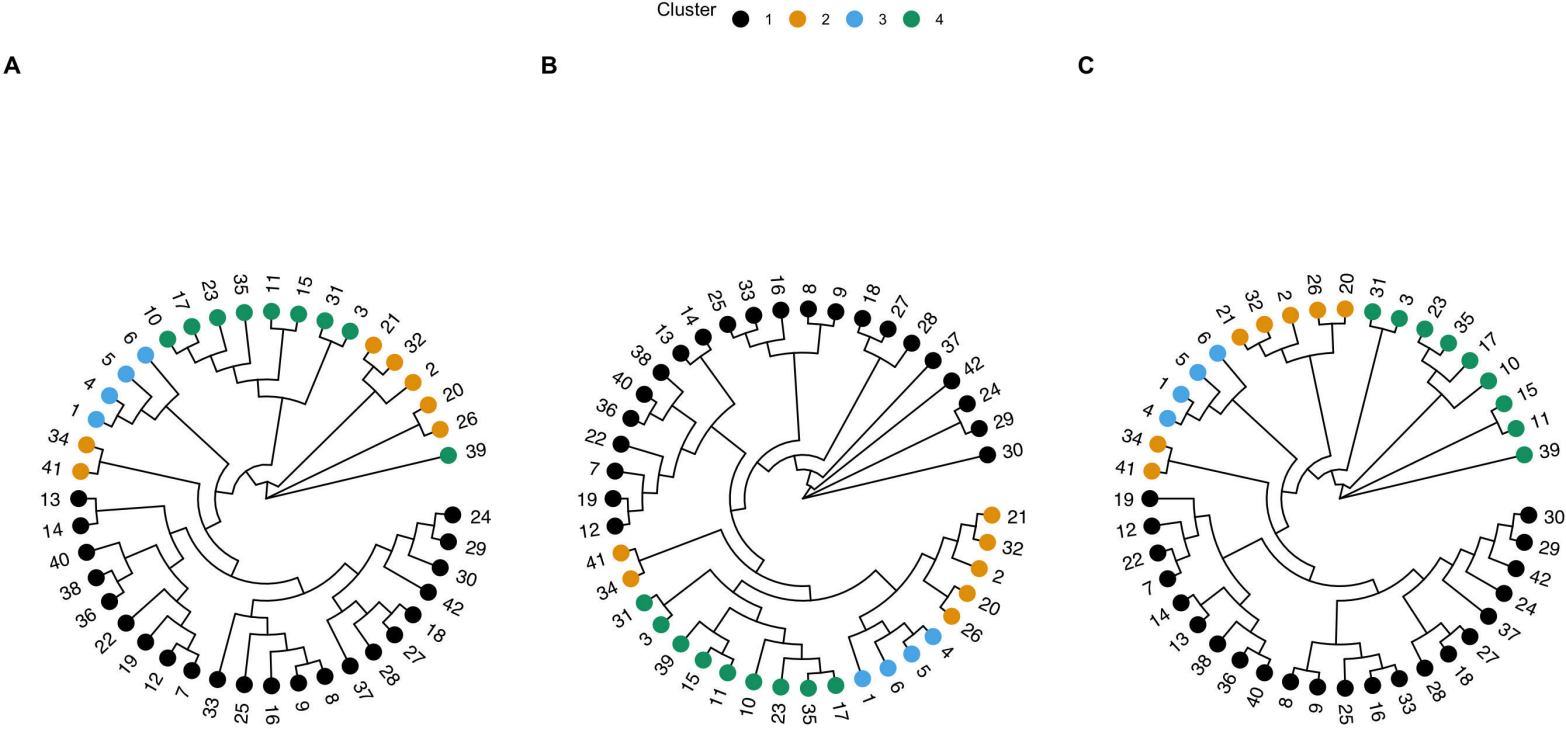
Circular genetic trees assembled for the *short* outbreak, including (**A**) neighbor typing predicted genetic tree using neighbor typing; (**B**) reference genetic tree created using *mashtree*; and (**C**) reference maximum-likelihood (ML) phylogeny created using *PanACoTA*. Tips are colored by cluster, as determined using *rhierBAPS*, and the clusters from the ML reference method are mapped onto the best match trees for comparison. An arbitrary sample number is labeled at the tips for ease of comparison of sample locations between trees.

**Fig 4:**
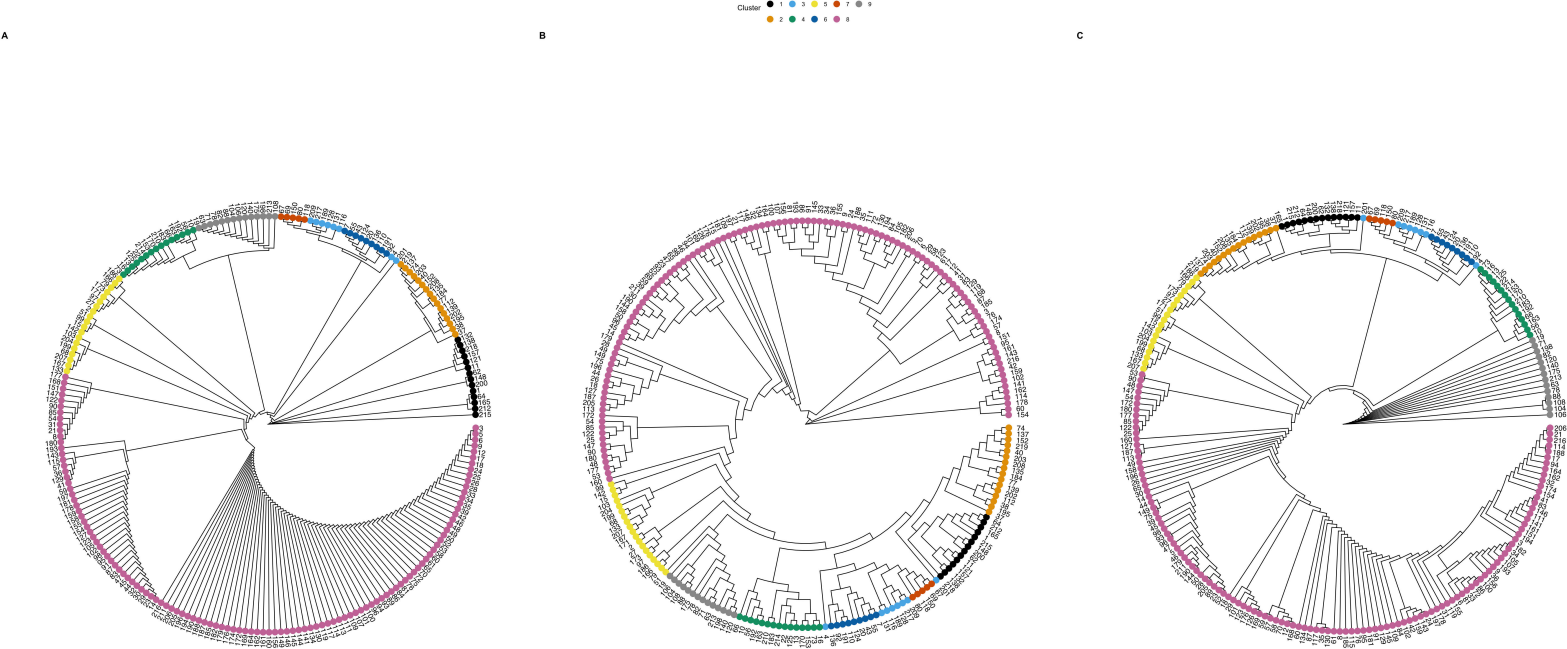
Circular genetic trees assembled for the *long* outbreak, including (**A**) neighbor typing predicted genetic tree using neighbor typing; (**B**) reference genetic tree created using *mashtree*; and (**C**) reference maximum-likelihood (ML) phylogeny created using *PanACoTA*. Tips are colored by cluster, as determined using *rhierBAPS*, and the clusters from the ML reference method are mapped onto the best match trees for comparison. An arbitrary sample number is labeled at the tips for ease of comparison of sample locations between trees.

We compared genetic trees (and clustering) generated using neighbor typing predictions with reference method trees including mash genetic trees and maximum-likelihood (ML) phylogenetic trees (Fig. 2 to 4). Overall, the trees generated by the neighbor typing method appeared largely consistent with those of both reference methods, where sample clustering was similar between predicted and reference approaches. In the *surveillance* data set, we observed seven distinct clusters (Fig. 2), with samples clustering similarly in the genetic tree (Fig. 2A) as in both of the reference tree/phylogeny (Fig. 2B and C). Samples were similarly clustered by MLST when that data were also paired with the tree (Fig. S9). Some discrepancies were observed within the *surveillance* data set (Fig. 2A), with samples 19 and 25, 23 and 50, and 6 and 10 having placement within the neighbor typing genetic tree that was not congruent with the reference trees/phylogeny. Sample 19, for example, belongs to cluster 5 but is placed within samples belonging to cluster 1 (Fig. 2A). For the 19/25 and 23/50 pairs, this apparent mismatch is likely due to one sample of each pair (19 and 50) having a best match with a discordant MLST, which results in misplacement within the tree.

Overall, this *surveillance* data set had a nucleotide diversity of 0.01397 (Table 1). We found a clustering comparability index (CCI) of 86% when comparing the neighbor typing genetic tree to the reference ML tree with hierBAPS clusters and computed a Baker’s gamma index (BGI) of 0.8 for both the neighbor typing genetic tree compared to the reference ML tree, as well as the neighbor typing genetic tree compared to the mash tree made using the reference samples (Table 1). Both measures indicate a high level of agreement between the neighbor typing-derived tree clustering and the reference ML tree clustering for the *surveillance* data set. The generalized Robinson-Foulds (GRF) distance for the neighbor typing genetic tree relative to both reference trees (0.63–0.64) suggests differences in topology and splits between the pairs of trees; however, this is not reflected in the cluster-focused metrics (CCI and BGI).

**TABLE 1:** Cluster comparability index (CCI), Baker’s gamma index (BGI), and generalized Robinson-Foulds (GRF) distance metrics for the three *Escherichia coli* data sets, including the *surveillance* database, and *short* and *long* outbreaks^*a*^^,^^*b*^

Data set	Number of clusters	Total samples	Nucleotide diversity	CCI	BGI		GRF distance	
				NT vs. RP/BAPS	NT vs. RM	NT vs. RP	NT vs. RM	NT vs. RP
Surveillance	7	51	0.01397	0.86	0.8	0.8	0.64	0.63
Short outbreak	4	42	0.01939	0.97	0.8	0.8	0.79	0.80
Long outbreak	9	218	0.01047	0.99	0.95	0.95	0.34	0.35

^*a*^ NT: Neighbor typing based genetic tree. RM: Reference tree created using *mashtree*. RP: Reference tree created using ML (*PanACoTA*).

^*b*^ The extent of clustering and the total samples are identified for each data set. All best matching isolates are identified as being concordantly or discordantly clustered relative to the tree created with the ML reference method.

The isolates in the *short* outbreak data set were clustered into four distinct clusters, and isolates again clustered similarly regardless of whether we evaluated the neighbor typing genetic tree (Fig. 3A) or either reference tree (Fig. 3B and C). We also observed clustering of the same MLSTs when that data were also paired with the tree (Fig. S10). In this data set, we found a 97% CCI for the neighbor typing genetic tree compared to the ML reference tree with hierBAPs clusters, and a BGI of 0.8 when comparing the genetic tree to both reference trees (Table 1). These values indicate a high level of agreement between the neighbor typing-derived tree clustering and the reference tree clustering. One obvious misclustering is with sample 39, which belongs to cluster 4, is found to be separate from the rest of the cluster using the neighbor typing genetic tree (Fig. 3). This data set had the highest nucleotide diversity with a value of 0.01939 (Table 1). As with the surveillance data set, the GRF values (0.79–0.80) suggest some difference in the topology and splits between the trees.

We next assessed the relatedness of the *long* outbreak isolates for every calendar year in the published set with iterative additions of the outbreak isolates to the database. We observed high similarity between the predicted and reference trees for isolates collected between 2010 and 2015 (Fig. S12 to S17). Following the final iterative addition of yearly isolates to the database, we assessed the final tree constructed using the best matches for all isolates from the database supplemented with the 2010–2015 surveillance samples, relative to the tree of the original isolates (Fig. 4). The *long* outbreak isolates in the final set were clustered into nine distinct groups, and isolates clustered similarly regardless of whether we assessed the genetic tree generated using the neighbor typing method (Fig. 4A) or either reference tree approach (Fig. 4B and C). As with the previous two data sets, isolates clustered similarly by MLST when this data were considered alongside the trees; however, there were some instances of non-monophyletic STs (Fig. S11). For example, ST 131, 401, and 410 were particular instances of non-monophyletic STs across the reference trees and the genetic tree derived from neighbor typing, with STs 216, 405, and 635 also being non-monophyletic in the neighbor typing genetic tree. For this final data set, we found that the genetic tree had a CCI of 99% when comparing the neighbor typing genetic tree to the ML tree and computed a BGI of 0.95 when comparing the genetic tree to both the mash and ML reference trees (Table 1). The GRF for this data set (0.34–0.35) suggested a greater similarity of topology (and splits) between tree pairs compared to the *surveillance* and *short* outbreak data sets (Table 1). This data set also had the lowest calculated nucleotide diversity with a value of 0.01047 (Table 1), and this reduced diversity may explain the improved BGI, CCI, and GRF similarity metrics for this data set over the other two data sets. This lack of diversity—a result of this data set containing more samples (*n* = 218) with greater opportunity for similarity (or clonality) than in the *surveillance* and *short* outbreak (*n* = 51) data sets—may have contributed to a greater ability of the neighbor typing methods to provide accurate clustering assignment and tree topologies.

## DISCUSSION

In this study, we evaluated an approach to rapidly assess the relatedness of antibiotic-resistant bacteria and potential transmission events using a combination of long-read sequence data paired with genomic neighbor typing. We found that predicted genetic distances derived from neighbor typing could be used as a proxy for reference method genetic distance measures between pairs of *E. coli* isolates/samples. We were also able to apply this method to recreate approximate genetic trees for two published outbreak data sets and recapitulated transmission clusters found using reference methods. Taken together, this provides evidence for the potential of this method to be utilized as a rapid surveillance tool.

Historically, WGS and the resulting draft genomes have been used as the gold standard method to help establish transmission events combined with other epidemiologic context when investigating outbreaks of AROs (15, 16). Due to the time and resources needed for WGS approaches (often taking days to generate results and requiring specialized bioinformatic expertise), they have traditionally been used as retrospective tools for the identification of outbreaks using cultured isolates, and less commonly for identifying potential transmission events during an ongoing outbreak (17–23). Prior studies have evaluated the use of metagenomic sequencing (direct from specimens without requiring a culturing step) for more rapid identification of potential nosocomial transmission events, rather than relying on the sequencing of isolates, and have shown the viability of metagenomic sequencing for outbreak surveillance (24, 25).

While the creation of the neighbor typing tree generally showed high agreement between resultant clusters and the reference trees, there were differences, which can be due to factors related to non-target metagenomic reads, diversity of reference databases, correspondence between isolates and primary specimens, and also errors with phylogenetic placement, which have been noted previously in the literature regarding likelihood-based tree methods (26). Similarly, there were some instances where clustering did not result in monophyletic sequence types (STs). MLST is not completely consistent when typing organisms, particularly in cases where recombination can impact the ability to accurately use MLST schemes to identify ST (26). However, we note that most of these instances occurred within the neighbor typing genetic trees, which are based on the best matches, which may not always be correctly assigned using RASE. Ultimately, the neighbor typing approach can be used in two ways, either to confirm an outbreak by identifying highly related samples or isolates (either through sequence typing or more in-depth analysis) or by ruling out an outbreak (by identifying that two or more samples are too genetically distinct to be related) (27). This work is most aligned with the latter and could be a particularly useful tool for ruling out potential transmission events, considering that identifying and confirming transmission would require more intensive analysis. However, we note that there have been recent major improvements to Nanopore sequencing technology and basecalling software which have been made available after this work was completed; improvements to the overall sequencing quality may translate into improved predictions if integrated into this workflow (28).

Considering that previous studies have shown that metagenomic sequencing can be useful for assessing relatedness, this has generally been limited to short-read sequencing (25). Here, we show that sequencing metagenomic samples using long-read technology could provide sufficient data to predict a best match, which is generally a good proxy for the true sample and can then be used to approximate the relatedness of that sample to the other samples. In particular, this approach can easily be implemented with only a few hundred long reads, which can be available within minutes of initiating sequencing (25). However, the composition of the metagenomic sample could result in longer sequencing times being required to obtain enough reads for accurate predictions. Additionally, due to low-read requirements to obtain quality results, there is an advantage of being able to heavily multiplex samples for sequencing in a single run; that is, since fewer reads are required per sample, multiplexing several uniquely barcoded samples on a single flow cell can serve to achieve adequate reads for analysis while also reducing the number of sequencing runs required to obtain these data (29). Taken together, this means that this method can be both cost and time efficient, making it appealing for routine use in clinical settings.

There are some limitations to this study. First, we have only evaluated this method with *E. coli*. Further prospective studies with *E. coli* and other pathogens will be essential to assess its utility for broader surveillance and clinical implementation, though mechanistically it is reasonable to believe this approach would hold true (30). Second, we simulated long reads for the *short* and *long* outbreaks using assemblies based on short-read sequencing, which may not accurately capture the realistic sequencing results for true long reads, particularly for regions with repeats (31). Third, it will be necessary to integrate the steps between determining a best match and identifying the degree of relatedness to other samples and identify samples for further investigation. Fourth, as shown by the improved performance with larger data sets in our study and consistent with previous work (13), the performance of the neighbor typing method and its ability to accurately find the nearest neighbor for any given sample will rely heavily on the database and whether it is representative of the population of isolates that could be circulating. Fifth, while the GRF distances suggest that there are topological differences between the trees, these values follow patterns similar to those found with the other metrics. That is, the *long outbreak* showed the best performance across any metric when comparing the neighbor typing tree to both reference trees for that data set, whereas both the *surveillance* and *short outbreak* data sets did not perform as well using the same metrics. Notably, the clustering-based metrics (BGI and CCI) may provide the greatest practical measure of similarity for clinical applications, as they are indicators of clustering that would be most relevant for outbreak evaluation. Conversely, GRF distances represent stricter tree comparisons, the results of which are more abstract and may be difficult to directly translate to clinical relevance. Sixth, rates of homologous and non-homologous recombination can vary within and between bacterial species and impact the organization of the genome (32). As such, recombination events that may happen quickly and rearrange large portions of the genome may sufficiently alter the sample genome such that prediction of the correct lineage may become more difficult. However, neighbor typing was shown to be robust in two species (*S. pneumoniae* and *N. gonorrhoeae*) where there is high recombination (12). Finally, we have demonstrated the generation of trees using an iterative reference database generation approach, which assumes that the ability to prospectively integrate samples into the approaches’ database(s), while potentially more technically challenging, can provide a clear benefit to surveillance.

In conclusion, we found that long-read sequence data, including those from metagenomic sampling, paired with a neighbor typing algorithm can predict relatedness of *E. coli* and facilitate the generation of representative genetic trees that are similar to reference methods. These results show that this method is a potential tool to rapidly generate genetic trees with relevant cluster information and estimate relatedness of clinical samples. Such a tool could drastically improve patient care by virtue of decreasing turnaround times for the assessment of outbreaks and transmission events. However, future work is necessary to: (i) streamline the analysis pipeline to facilitate more rapid implementation; (ii) validate this approach with other ESKAPE pathogens; and (iii) identify ways to handle multiple organisms in a primary specimen.

## MATERIALS AND METHODS

### Study design

We performed a retrospective genomic evaluation of *k*-mer based neighbor typing for predicting genetic relatedness of *E. coli* compared to reference standard methods. We used real-world and simulated clinical and outbreak surveillance data from three previously published studies representing diverse geographic locations, time frames, and settings. Approval for this study was obtained in Ottawa by the Ottawa Hospital Science Network (#20200108-01H) and in Toronto by the Sinai Health (20-0161-E) and University Health Network (#20-5677) Research Ethics Boards.

### Study populations

We evaluated relationships between genetic relatedness from short read sequencing compared with *k*-mer based (RASE) analyses using long-read sequence data from: (i) a previously published study of Nanopore sequenced primary clinical samples containing antibiotic resistant and non-antibiotic resistant *E. coli* collected from critical care patients (*n* = 51; “*surveillance data set*” (13); and (ii) synthetic long-reads generated from two previously published studies of ARO and non-ARO *E. coli* outbreaks (“*short outbreak*,” *n* = 43 [33]; “*long outbreak*,” *n* = 268 [33]) (see supplemental methods for details) (17, 33). When referring to the samples within each data set, samples originating from the *surveillance* data set will be referred to as the “metagenomic samples,” as they are derived from sequencing the metagenomic content of the collected samples prior to filtering for *E. coli*-specific reads. Samples originating from the *short* and *long* outbreak data sets will be referred to as “isolates” originating from their respective study, as the reads originated from assemblies derived from isolate sequencing.

### Generation of the RASE database for assessing relatedness

Using RASE (v.1.0.0.0) (34) to create the neighbor typing database used for this study, we included isolates previously used for an *E. coli* database (*n* = 148) using MLST to identify lineages (13), and supplemented with additional genomes (*n* = 54) from EnteroBase in order to increase the diversity and representation of clinical *E. coli* STs (35). This new database consisted of 202 isolates and 91 STs (Table S1).

### Long reads for surveillance, short, and long outbreak data sets

For the *surveillance* data set, we used pathogen-specific reads generated by Nanopore sequencing, followed by filtering using *Kraken2* (v.2.1.3) with standard RefSeq database (k2_standard_20230605 database) (36) and *KrakenTools* (v.1.2) (37). For both the *short* and the *long* outbreaks, we used simulated Nanopore reads created using the reference available draft genomes and *nanosim-h* (v.1.1.0.4) using the included default *E. coli* error profile (ecoli_R9_2D) (38) (see supplemental methods for more details). For the outbreak data sets, only 500 simulated reads were generated per isolate.

### Predicting MLST and relatedness using neighbor typing

Each prediction by neighbor typing produces a “best match” isolate to a single genome in the neighbor typing database using long-read (real or synthetic) sequencing data, as well as the MLST of the best matching isolate (38). The best match can also be used to determine the genetic distance of the query sample to other samples based on their respective best-matching isolates.

### Evaluating the relationship between neighbor typing predicted genetic distance and reference genetic distance

Pairwise genetic distances were calculated between all isolates in the neighbor typing database(s). These differences were used as surrogate distances between best match isolates, which were identified using the neighbor typing approach. Henceforth, we will be referring to the genetic distances obtained from the maximum likelihood tree created using PanACoTA on the paired isolates for the samples we queried against the neighbor typing database to obtain a best match as the “reference standard” genetic distance(s). Neighbor typing predicted genetic distances (including SNP differences) were plotted against the reference standard genetic distances determined using the short-read data from draft genomes. Predicted and reference standard differences were plotted and non-parametric measures of correlation (Spearman) were determined using R (v.4.3.0 (39) and *ggplot2* (v.3.5.1) (40, 41). Bootstrapped Spearman’s rho was determined using *rcompanion* (v.2.5.0)(41). We also stratified the results for samples with LS ≥ 0.5, which can indicate samples with higher confidence in the neighbor typing match.

### Comparing neighbor typing generated genetic trees with reference standard mash genetic and ML phylogenetic trees

We then sought to compare the genetic trees created using the best matches generated using neighbor typing to two trees created using the reference standards. For the reference standards, two methods were utilized to create the trees, *mashtree* (a neighbor-joining tree method) (42) and ML with *PanACoTA* (43). These represented trees are non-rooted trees, and the radial distances are not directly proportional to the genetic distances between isolates. See Supplemental Methods for further details.

### Clustering analysis

We sought to quantify the comparability of clustering between the neighbor typing predicted genetic tree and the two reference trees (mash and ML). We used three metrics: the BGI (44), the GRF distance (45), and the last being a statistic we devised, which we term the cluster comparability index (CCI). 95% CIs were also evaluated for the CCI. See Supplemental Methods for further details.

### Applying an iterative database generation approach to outbreak evaluations

In order to assess the ability of our method to construct a database progressively, where samples from surveillance could routinely be added to an updated database to match highly related isolates that may have been transmitted. We used isolates from the *long* outbreak data set and iteratively added isolates from each calendar year reported in the study (2010–2015) into a new neighbor typing database and assessed the resulting predictions as samples from additional years were added to the database. We describe this further in the Supplemental Methods. See Fig. S1 for a graphical overview of the neighbor typing method described here.

## Data Availability

Data used are available as referenced in the cited publications (13, 17, 33). The updated RASE database(s) are available on Zenodo (DOI: 10.5281/zenodo.15684054). The filtered FASTQ files used for the surveillance data set are available on Zenodo (DOI: 10.5281/zenodo.15684101); note that these FASTQ files are filtered for *E. coli*-specific reads and include reads only until the point of stability.

## SUPPLEMENTAL MATERIAL

Supplemental material aac.01071-25-s0001.docx Supplemental methods; Fig. S1 to S17; Tables S1 and S2.

ASM does not own the copyrights to Supplemental Material that may be linked to, or accessed through, an article. The authors have granted ASM a non-exclusive, world-wide license to publish the Supplemental Material files. Please contact the corresponding author directly for reuse.
