# Supplementary material for: Prediction of genetic relatedness of Escherichia coli using neighbor typing: a tool for rapid outbreak detection
Source: Antimicrob Agents Chemother. 2026 Jan 26;70(3):e01071-25. doi: 10.1128/aac.01071-25 (PMC12959140; doi:10.1128/aac.01071-25)
Supplement: Supplemental material — Supplemental methods; Fig. S1 to S17; Tables S1 and S2. [file aac.01071-25-s0001.docx]

**Supplemental Materials and Methods**

***Supplemental Methods***

*Study Populations - Extended*

For study (1), termed *surveillance*, the 51 primary clinical samples were urine and respiratory specimens collected from critical care patients at four tertiary care hospitals in Ontario in 2021 confirmed to contain *E. coli* as the dominant Gram-negative pathogen that were directly sequenced using the Oxford Nanopore MK1c. Study (2), termed *short outbreak*, represented a temporally limited outbreak that included 43 *E. coli* isolates out of 155 total isolates collected from neonates as part of routine screening at City Hospital, Birmingham UK [(Price et al. 2022)](https://paperpile.com/c/a2agwD/v8pVb). The third study (3), termed *long outbreak*, represented a multi-year outbreak, which was investigated as part of a surveillance program of KPC-producing *E. coli* that consisted of 268 sequenced isolates from screening samples collected between January 1, 2010 to January 1, 2017 in the Manchester Heart Centre (MHC) at the Central Manchester University Hospital NHS Foundation Trust [(Decraene et al. 2018)](https://paperpile.com/c/a2agwD/jmNt8).

*Evaluating the Relationship Between Neighbour Typing Predicted Genetic Distance and*

*Reference Genetic Distance* - Extended

We also compared the genetic distances between methods by MLST concordance between sample pairs, as well as the lineage score (LS, a measure of prediction confidence produced by RASE for each predicted best match), using a previously evaluated threshold of ≥ 0.5 as a measure of a confident lineage prediction. Since we assessed the concordance of two pairs of samples in each case, samples ultimately labelled “concordant” had a concordant MLST best match for both test samples; pairs labelled “discordant” had either one or both best match(es) having a discordant MLST to their test sample(s).

*Predicting MLST and Relatedness using Neighbour Typing - Extended*

We evaluated a best match by identifying a “stable call”, as described in our previous work, whereby the lineage score did not change by more than ±0.1 for 100 reads, and found a median of 203 (IQ1:161 - IQ3: 345) was necessary to make a stable call for *E. coli* using the surveillance dataset [(Carroll et al. 2024)](https://paperpile.com/c/a2agwD/Lsrdi).

Using draft genome assemblies created using short-read sequencing data, PanACoTA (v.1.4.1) [(Perrin and Rocha 2021)](https://paperpile.com/c/a2agwD/O1Mcj) was utilised to create the pairwise genetic distances for the neighbour typing database isolates (pool of potential best matching isolates), as well as to determine the reference pairwise genetic distances for all of the query samples.

*Creation of Synthetic Nanopore Reads*Nanopore reads were not available for analysis for either published outbreak dataset, so they were simulated using NanoSim-H with default error settings (insertion/deletion/mismatch rate tuning left on default of [1.0] using the profile (ecoli_R9_2D) for *E. coli*), but limited read lengths to a minimum of 200 bp and maximum of 10,000 bp, and only generated 500 reads per sample [(Yang et al. 2017; “Karel-brinda/NanoSim-H: NanoSim-H 1.1.0.4,” n.d.)](https://paperpile.com/c/a2agwD/gOcEJ+QHHGb) using the fasta file from the assembly of each isolate. Isolates without a published assembly were omitted from analysis. We found an average read length of 3512bp for the *surveillance* dataset, while the *short* outbreak dataset had an average read length of 6042bp and the *long* outbreak had an average read length of 6003bp. The simulated reads were then run through the neighbour-typing algorithm and the best matches recorded to be used for further analysis.

*Assessment of Samples in the Surveillance Isolate Dataset*

Four pairs of samples were not included in the *surveillance* dataset where there was a possibility of mis-labelling identified during processing.

*Comparing Neighbour Typing Generated Genetic Trees with Reference Standard Mash Genetic and Maximum-Likelihood (ML) Phylogenetic Trees - Extended*

*Mashtree* was used with standard parameters on all genome assemblies for the reference standards for each of the *surveillance*, *short* outbreak, and *long* outbreak datasets. *PanACoTA* was used with standard parameters for all steps except the annotation step, which was run with the –prodigal flag and where –l90 was adjusted to 999 (default 100) and –nbcont was adjusted to 3500 (default 999) to include all genomes previously contained within the original neighbour typing reference database. For the neighbour typing predicted genetic tree, we used the genome assemblies for the best matching isolates as determined from the neighbour typing database, and then used the same parameters in *PanACoTA* to create the genetic tree. Again, these best matching isolates are chosen on the basis of determining the best match for each sample or isolate using the long reads, and the tree representing how these samples appear to be related based on those best matches is created using core genome alignment from the genome assemblies of those best matching isolates, as is done with the reference standard isolates. Using the core genome produced in *PanACoTA* for each dataset’s reference standard isolates, we used *rhierBAPS* (v.1.1.4) with a max.depth of 2 and n.pops of 20 [(Kassambara 2017; Cheng et al. 2013)](https://paperpile.com/c/a2agwD/jsq2p+ceP0C) to determine the clustering of the isolates (at the first cluster level). Clustering information was then used for highlighting clustered samples on the reference standard ML phylogeny using *ggtree* [*(Yu 2022)*](https://paperpile.com/c/a2agwD/ehnL6) as described [(Tonkin-Hill et al. 2018; Cheng et al. 2013)](https://paperpile.com/c/a2agwD/iIX27+ceP0C). These represented trees are non-rooted trees and the radial distances are not directly proportional to the genetic distances between isolates.

*Cluster Analysis - Extended*
Considering the maximum likelihood phylogeny with hierBAPS as a reference standard depiction of clustering, we identified each of the individual clusters within a dataset. We then sought to quantify to what extent clustering was comparable (or consistent) with this phylogeny, for both the mash and predicted genetic trees, using a metric we call the cluster comparability index (CCI). If a cluster contained six isolates, and each were grouped together, we assume that each of those isolates should be grouped with the five other individuals in the cluster, meaning that each of those isolates would get a score of 5/5 for each being grouped with the five isolates as expected; the total (sum) score for the cluster would then be 6, as each of the six isolates have a score of 5/5, or 1. If a second cluster had nine isolates total, and was grouped into two distinct regions of the overall tree, one with five isolates and one with four isolates, we would expect each of the isolates in the cluster to be grouped with the eight other isolates, and score the two regions as follows: in the first grouping of five isolates, each is scored as 4/8, as each isolate is now only grouped with four other isolates, rather than all eight ((4/8)*5 isolates=2.5), and the second group would have each isolate scored as ⅜, as each isolate is now grouped with only three of the eight other isolates it should be grouped with ((⅜)*4 isolates=1.5). If this tree only contained the two described clusters, we would then calculate the total cluster concordance (sum of the score for each cluster/total number of isolates) as 0.667 ((2.5+1.5+6)/15). The 95% confidence intervals were then calculated as follows: margin of error = 1.96*sqrt ((p*(1-p))/n), where *p* is the proportion of correctly clustered isolates, *n* is the number of samples in the tree to assess, and 1.96 is the z-score for the 95% confidence interval. Using the above example, we calculate the margin of error as 1.96*sqrt((0.667*(1-0.667))/15), and get a value of 0.238. This results in a CCI confidence interval of 0.667 (95% CI: 0.429, 0.905).

To calculate the BGI, the genetic distance matrix for each tree was imported and processed using *dendextend* [*(Galili 2015)*](https://paperpile.com/c/a2agwD/61AHT) in R, and the Baker’s gamma index computed for each pair of trees in each dataset. Following the import of the matrix, the as.dist() function was used to ensure the data was appropriately formatted. hclust() was then used for hierarchical clustering, followed by as.dendro() to create the relevant dendrogram. Cor_bakers_gamma(dendrogram1, dendrogram2) was used to evaluate the Baker’s gamma index for the evaluated pair. To calculate the generalized Robinson-Fould distances for each tree pair, the trees were imported into R and processed with *TreeDist* (v.2.11.1) using MutualClusteringInfo [(Smith 2020)](https://paperpile.com/c/a2agwD/zLGt2). The CCI was devised as a measure of how often samples share the same cluster neighbors in a new genetic tree compared to hierarchical clusters (hierBAPS) determined from reference draft whole genomes, and results in a proportion that ranges from 0-100% (See supplemental materials for calculation details). In contrast to the BGI, the CCI was only calculated for neighbour typing predicted trees and compared with hierBAPS (level 1) clusters. We note here that neighbour typing predicted trees and the reference standard trees created using *mashtree* will be identified as “genetic trees” throughout, whereas “phylogeny” will be used to describe the reference standard tree created using the core genome and *PanACoTA*.

*Lineage Score Assessment*

To confirm that there were no major discrepancies for the lineage scores (LS) produced by the simulated reads for the two published datasets and the reads directly generated with long-read sequencing, we plotted a histogram to observe the distribution of LS (Supplemental Figures 6 and 7).

*Applying an Iterative Database Generation Approach to Outbreak Evaluations - Extended*

That is, using our described baseline neighbour typing database, we applied the approach for all samples in the *short* outbreak or the Year 1 (2010) samples for the *long* outbreak [(Carroll et al. 2024)](https://paperpile.com/c/a2agwD/Lsrdi). For the isolates for the remaining years of the *long* outbreak, we updated the reference database with isolates from Year 1, and applied the neighbour typing approach with samples from Year 1 and Year 2. We then applied this iterative approach to each subsequent year of samples (see Supplemental Table 2 for details on which samples were included in each database iteration, and which samples were queried). With the final iteration of the database and sample analysis, we created the appropriate genetic or reference trees with clustering, as described above.

*Overview of Method*


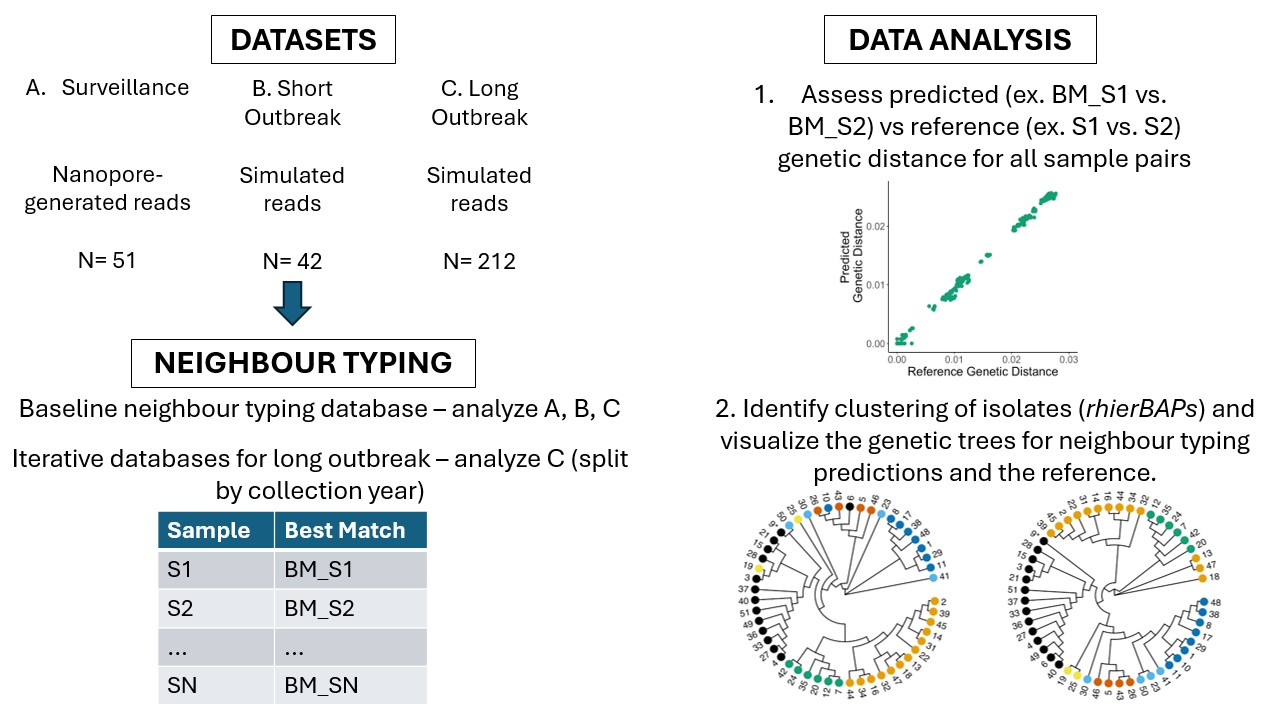


Supplementary Figure 1: Overview of the neighbour typing method used to predict the relatedness of isolates from three *E. coli* datasets.

[Alt text: Graphical representation of the neighbour typing method used for predicting the relatedness of the isolates in three *E. coli* datasets. The figure is separated into three main sections, one giving an overview of the three datasets, followed by a sample of the main output from neighbour typing predictions. The final section represents a sample of the results from the two main analyses (relating predicted genetic distances generated using neighbour typing to reference genetic distances, and visualizing the genetic tree created using the neighbour typing method), as well as text summarizing the Methods section for how those results were generated.]

***Supplemental Figures***


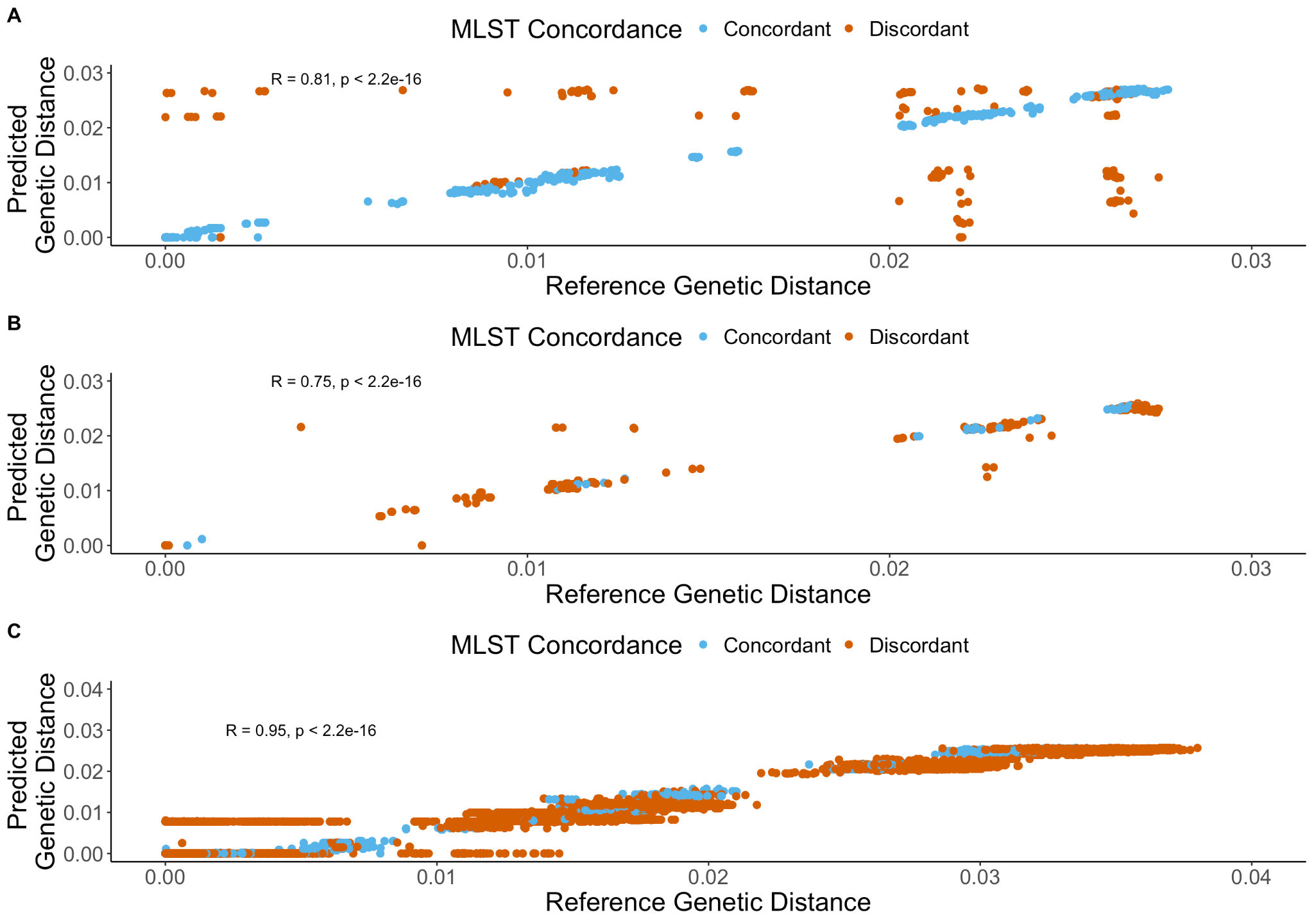


Supplementary Figure 2: Plot of reference and predicted genetic distances for *E. coli* datasets using all samples without stratification. (A) represents *surveillance* dataset, (B) represents the *short* outbreak, and (C) represents the *long* outbreak. Blue data points are predictions for concordant calls, and orange data points are predictions based for discordant calls.

[Alt text: Graphs comparing the predicted genetic distance to the reference genetic distance of samples or isolates in three datasets with subfigures labelled from A-C. Clear linear relationships are shown for each dataset, although not as strongly as Figure 1.]


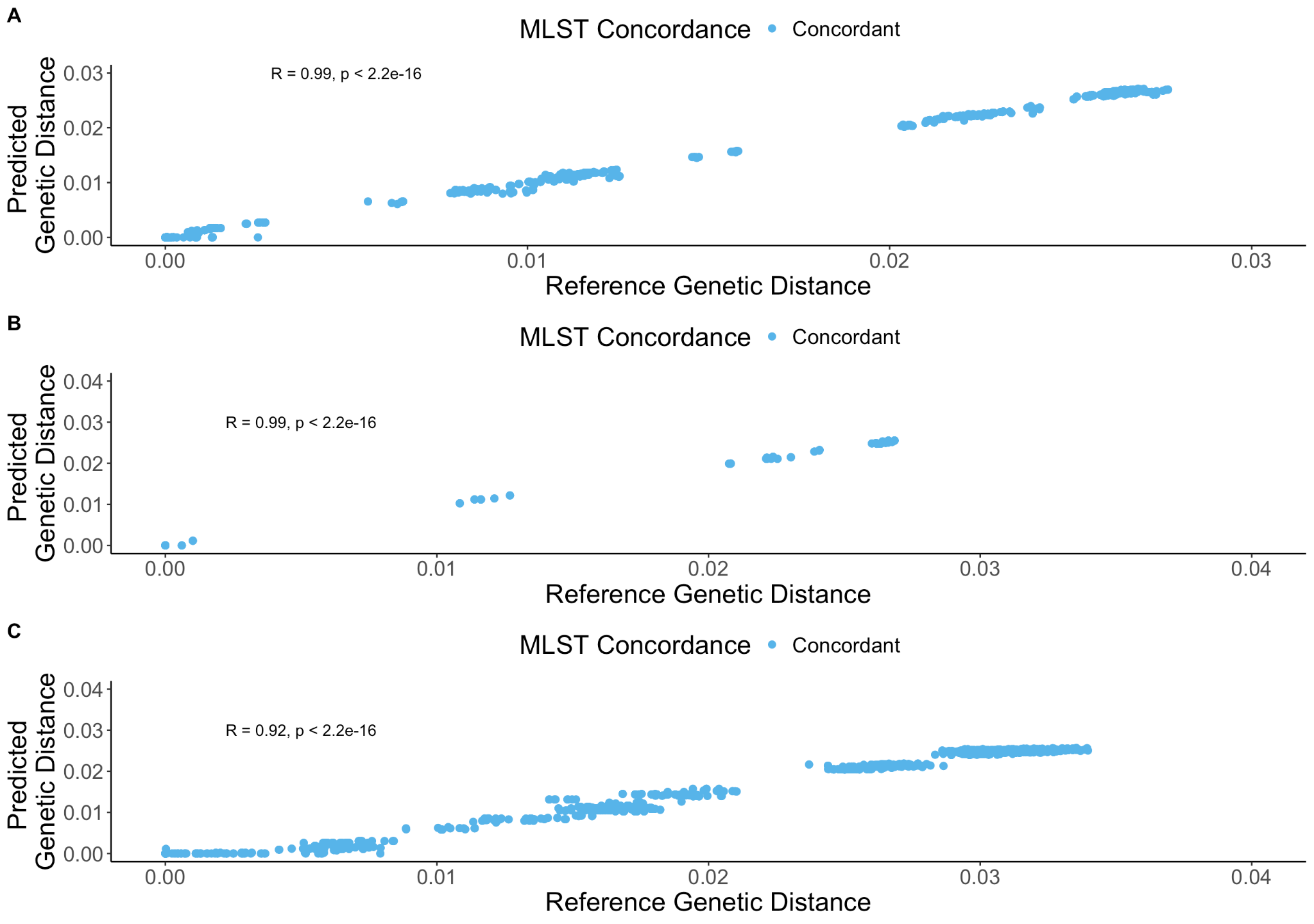


Supplementary Figure 3: Plot of reference and predicted genetic distances for *E. coli* using only MLST concordant samples without stratification. (A) represents *surveillance* dataset, (B) represents the *short* outbreak, and (C) represents the *long* outbreak. Blue data points are predictions for concordant calls, and orange data points are predictions based for discordant calls.

[Alt text: Graphs comparing the predicted genetic distance to the reference genetic distance of only MLST concordant samples or isolates in three datasets with subfigures labelled from A-C. Strong linear relationships are shown for each dataset.]


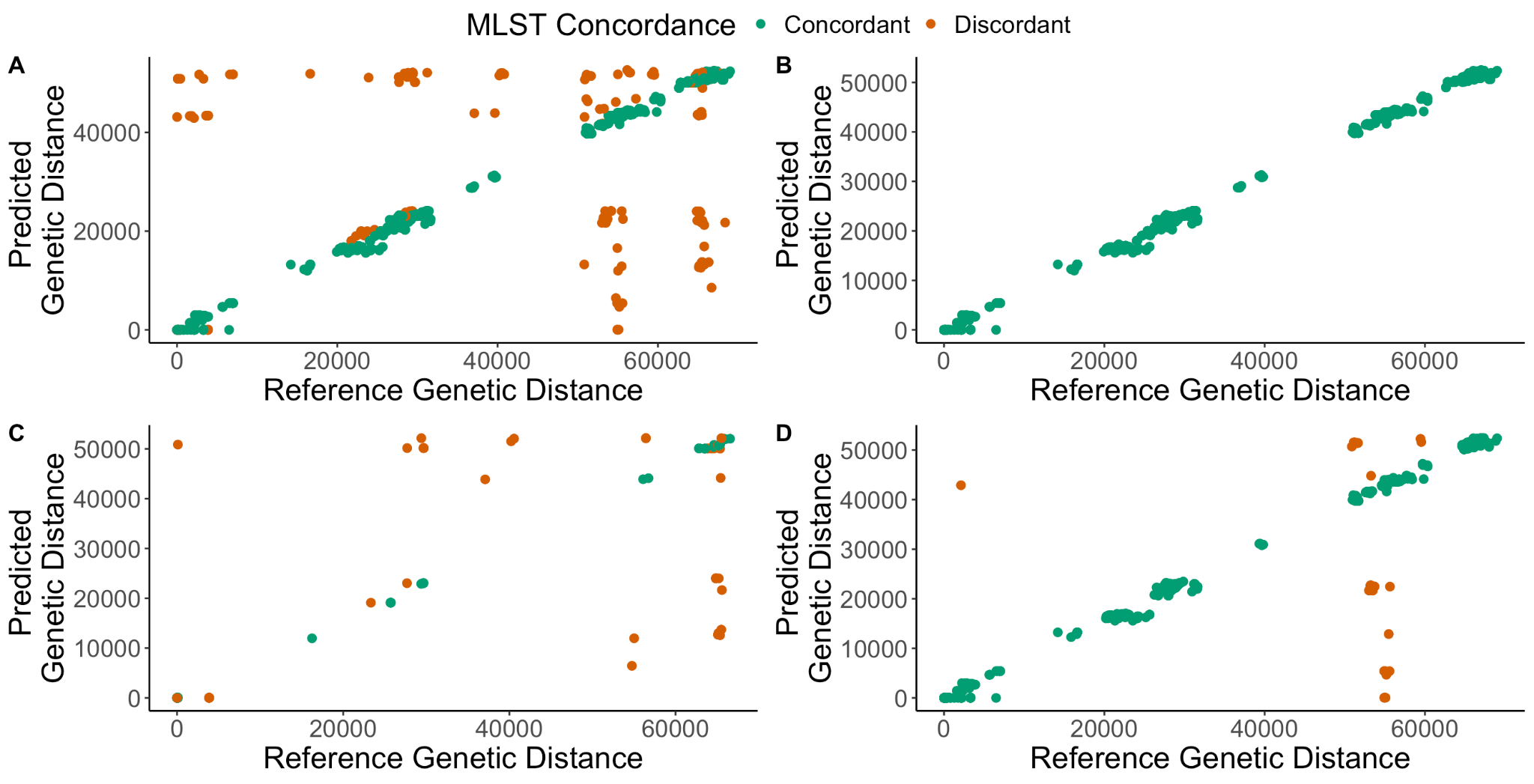


Supplementary Figure 4: Plot of reference and predicted SNP distances for *E. coli* for *surveillance* dataset. (a) All comparisons. (B) Only concordant comparisons. (C) All comparisons with LS<0.5. (D) All comparisons with LS>0.5. Green data points are predictions for concordant calls, and orange data points are predictions based for discordant calls. Note: this figure represents the same data in A, B, and D as presented in Figure 1A, Supplementary Figure 2A, Supplementary Figure 3A, but represents the SNP, rather than genetic, distances.

[Alt text: Graphs comparing the predicted SNP distance to the reference SNP distance for four data stratification levels of the surveillance dataset, with subfigures labelled from A-D. Strong linear relationships are shown for each subfigure.]


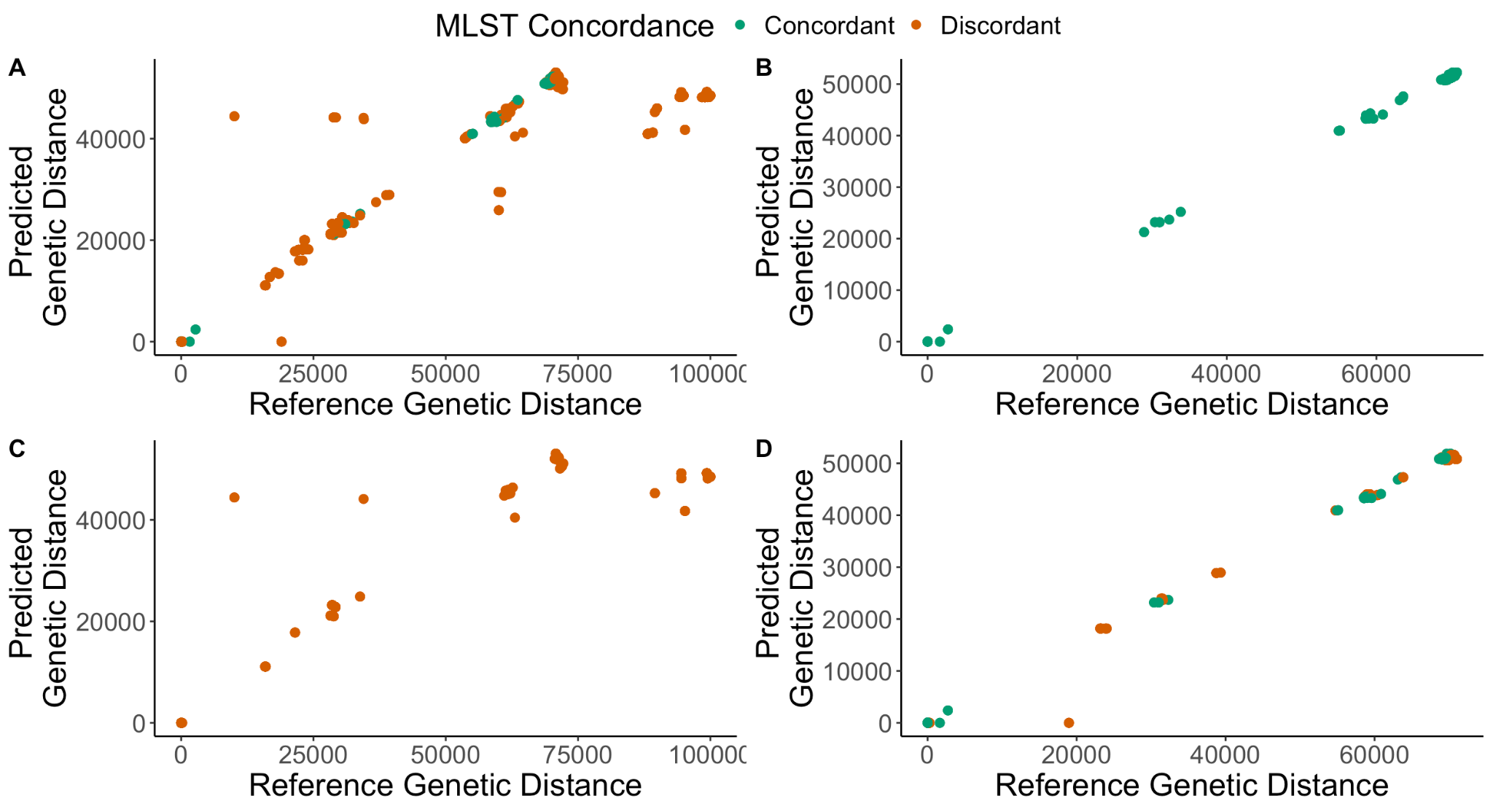


Supplementary Figure 5: Plot of reference and predicted SNP distances for the *short* *E. coli* outbreak dataset from. (a) All comparisons. (B) Only concordant comparisons. (C) All comparisons with LS<0.5. (D) All comparisons with LS>0.5. Green data points are predictions for concordant calls, and orange data points are predictions based for discordant calls. Note: this figure represents the same data in A, B, and D as presented in Figure 1B, Supplementary Figure 2B, Supplementary Figure 3B, but represents the SNP, rather than genetic, distances.

[Alt text: Graphs comparing the predicted SNP distance to the reference SNP distance for four data stratification levels of the short outbreak dataset, with subfigures labelled from A-D. Strong linear relationships are shown for each subfigure.]


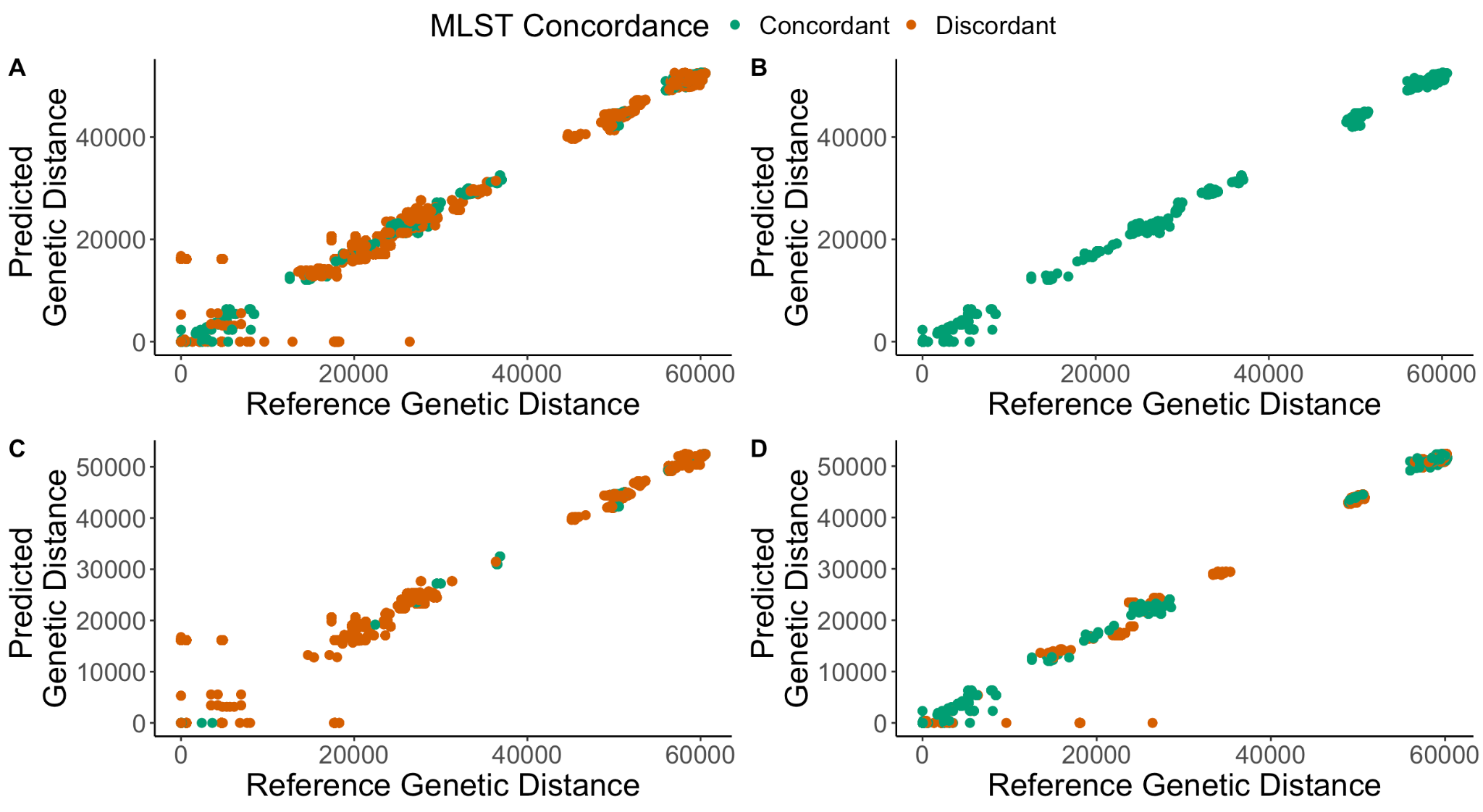


Supplementary Figure 6: Plot of reference and predicted SNP distances for the *long* *E. coli* outbreak dataset from. (a) All comparisons. (B) Only concordant comparisons. (C) All comparisons with LS<0.5. (D) All comparisons with LS>0.5. Green data points are predictions for concordant calls, and orange data points are predictions based for discordant calls. Note: this figure represents the same data in A, B, and D as presented in Figure 1C, Supplementary Figure 2C, Supplementary Figure 3C, but represents the SNP, rather than genetic, distances.

[Alt text: Graphs comparing the predicted SNP distance to the reference SNP distance for four data stratification levels of the *long* outbreak dataset, with subfigures labelled from A-D. Strong linear relationships are shown for each subfigure.]

**
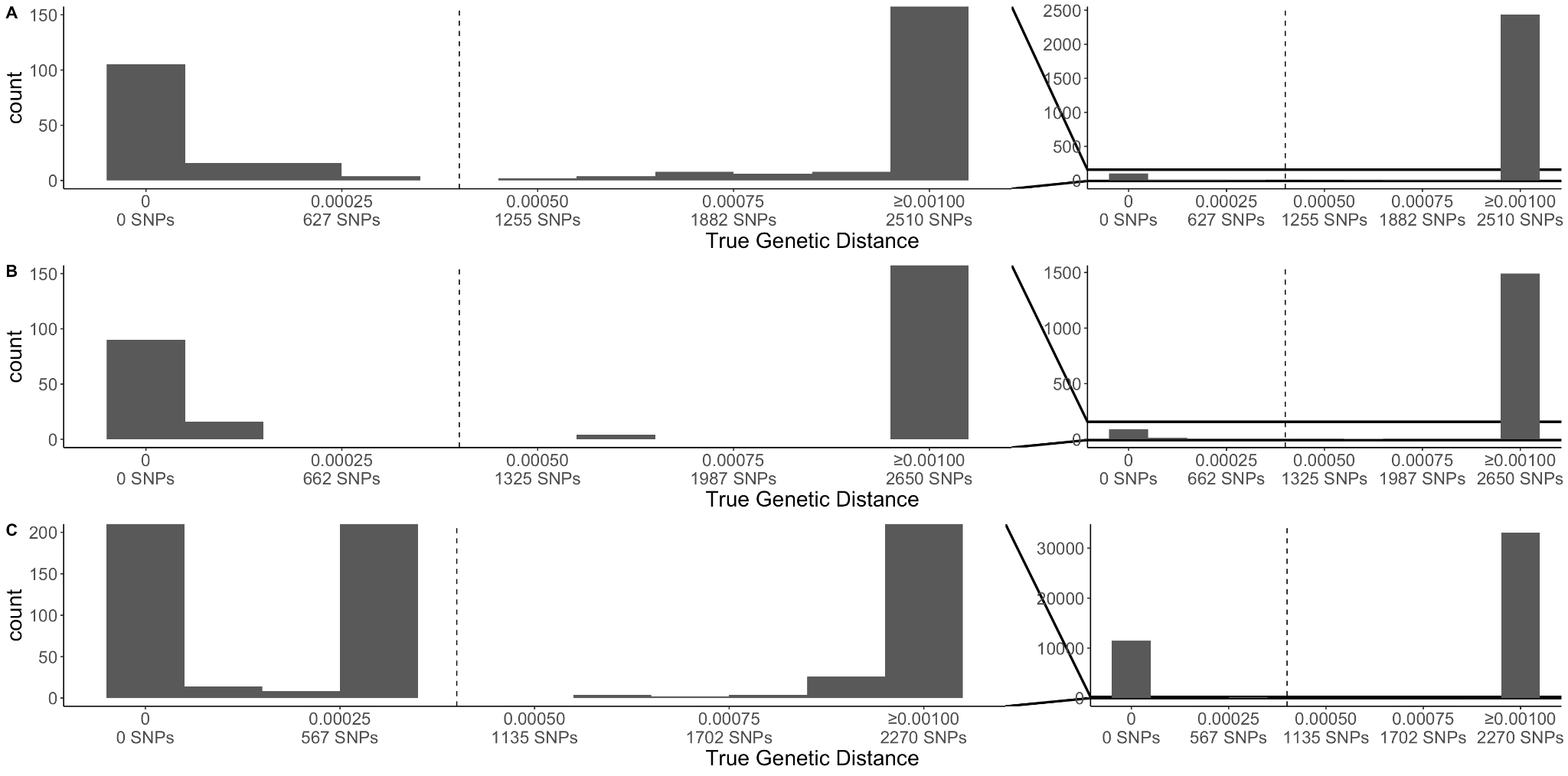
**

Supplementary Figure 7: Histogram of reference genetic distances for all three datasets. The x-axis represents the genetic distances of samples/isolates, and the count on the y-axis reflects the number of samples with values falling into the range of values in that bin. (A) *Surveillance* dataset. (B) *Short* outbreak. (C) *Long* outbreak. Horizontal dashed line marks a genetic distance of 0.000375.

[Alt text: Graphical representation of the reference genetic distances for the three datasets, with subfigures labeled A-C. Subfigures show a clear separation of samples with low genetic distances and those with larger genetic distances.]


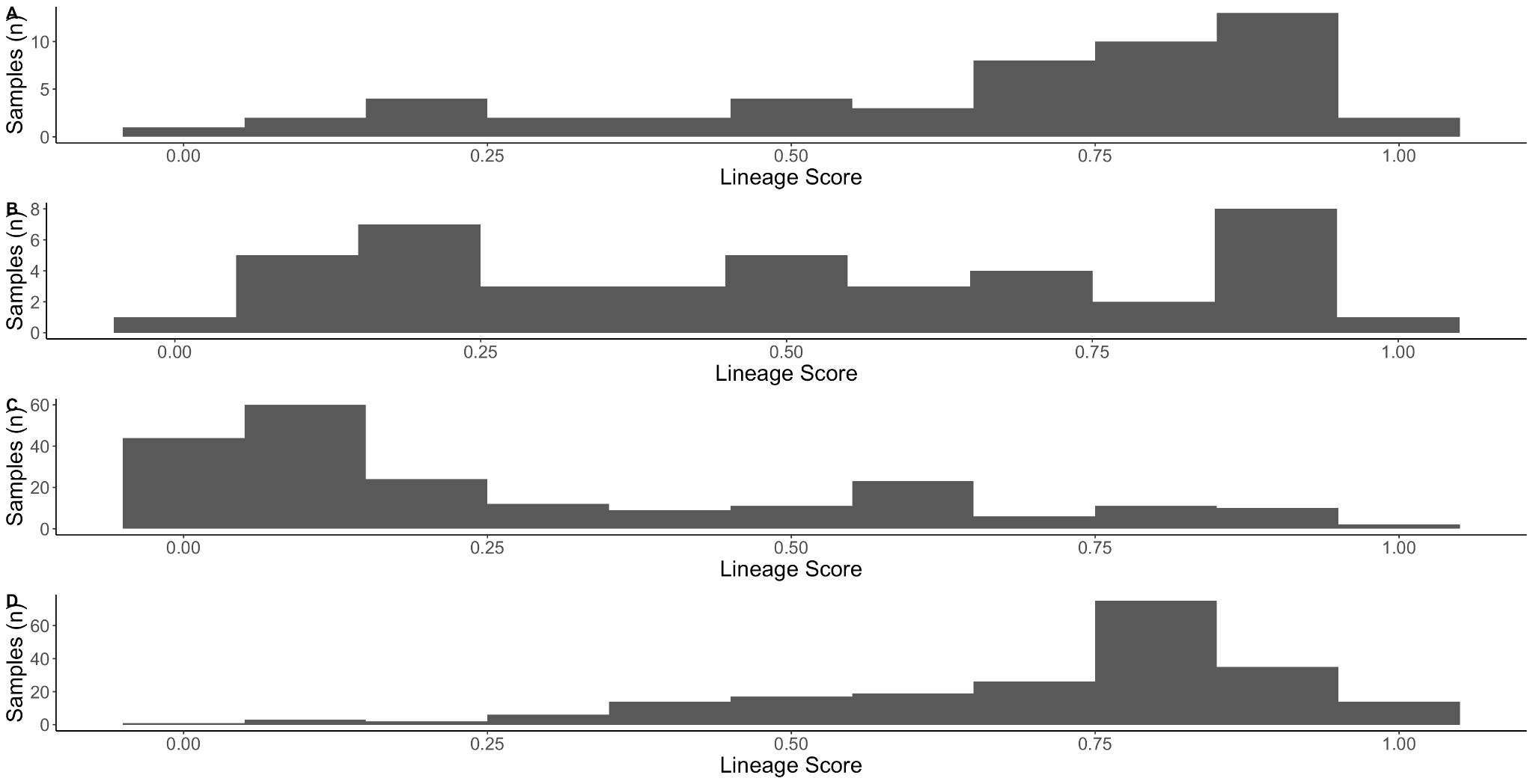


Supplementary Figure 8: Histogram of the distribution of lineage scores (LS) for all samples in each of the datasets. (A) Distribution of LS for the *surveillance* dataset. (B) Distribution of LS for the *short* outbreak[(Price et al. 2022)](https://paperpile.com/c/a2agwD/v8pVb). (C) Distribution of LS for the *long* outbreak [(Decraene et al. 2018)](https://paperpile.com/c/a2agwD/jmNt8) using the unsupplemented database. (D) Distribution of LS for the *long* outbreak from [(Decraene et al. 2018)](https://paperpile.com/c/a2agwD/jmNt8) using the final database supplemented with Years 1-5 from the outbreak dataset.

[Alt text: Graphical representation of the distribution of lineage scores, with subfigures labeled A-D. Graphs show different distributions of lineage scores for the different datasets.]


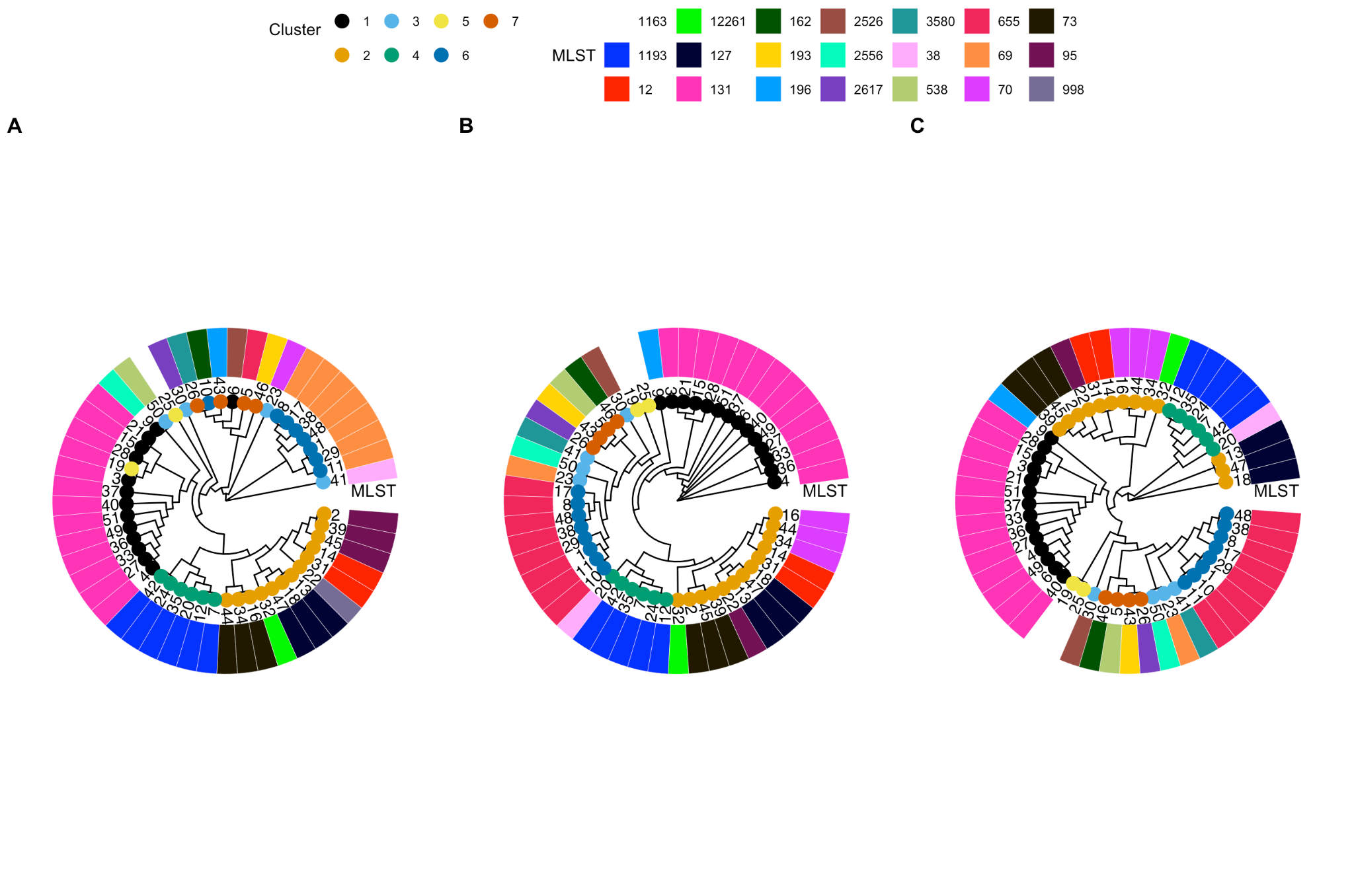


Supplementary Figure 9: Circular genetic trees assembled for the *surveillance* dataset including: (A) neighbour typing predicted genetic tree using neighbour typing; (B) reference genetic tree created using *mashtree*; (C) reference ML phylogeny created using *PanACoTA*. The outer ring denotes the MLST of each sample. Tips are coloured by cluster, as determined using *rhierBAPS*, and the clusters from the ML reference method are mapped onto the best match trees for comparison. An arbitrary sample number is labelled at the tips for ease of comparison of sample locations between trees.

[Alt text: Graphical representation of two genetic trees and one phylogeny for the surveillance dataset, with subfigures labelled A-C. This is an updated version of Figure 2, with an additional heatmap layer describing MLST of the sample. Samples generally cluster by MLST, regardless of tree.]


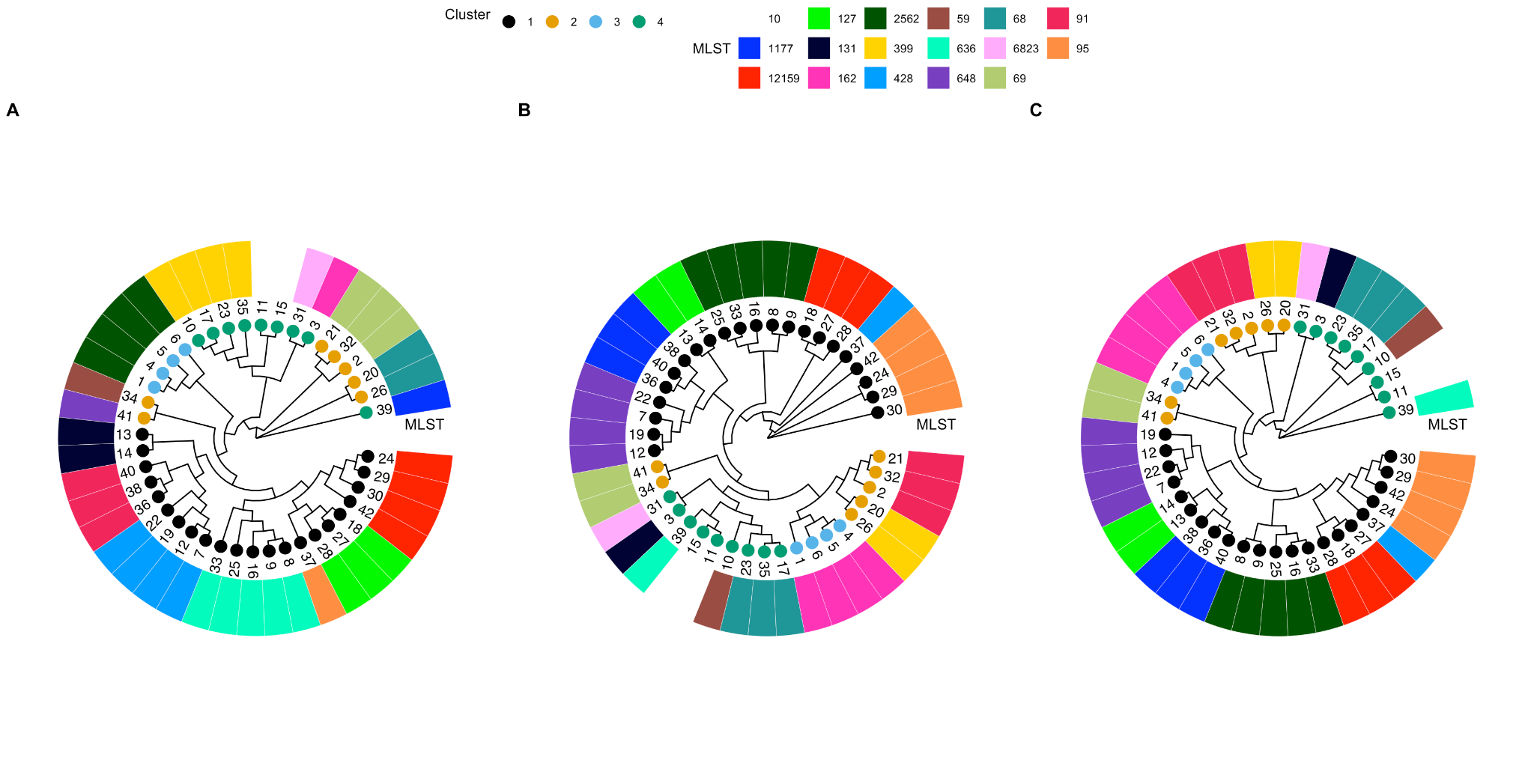


Supplementary Figure 10: Circular genetic trees assembled for the *short* outbreak including: (A) neighbour typing predicted genetic tree using neighbor typing; (B) reference genetic tree created using *mashtree*; (C) reference ML phylogeny created using *PanACoTA*. The outer ring denotes the MLST of each sample. Tips are coloured by cluster, as determined using *rhierBAPS*, and the clusters from the ML reference method are mapped onto the best match trees for comparison. An arbitrary sample number is labelled at the tips for ease of comparison of sample locations between trees.

[Alt text: Graphical representation of two genetic trees and one phylogeny for the *short* outbreak dataset, with subfigures labelled A-C. This is an updated version of Figure 3, with an additional heatmap layer describing MLST of the sample. Samples generally cluster by MLST, regardless of tree.]


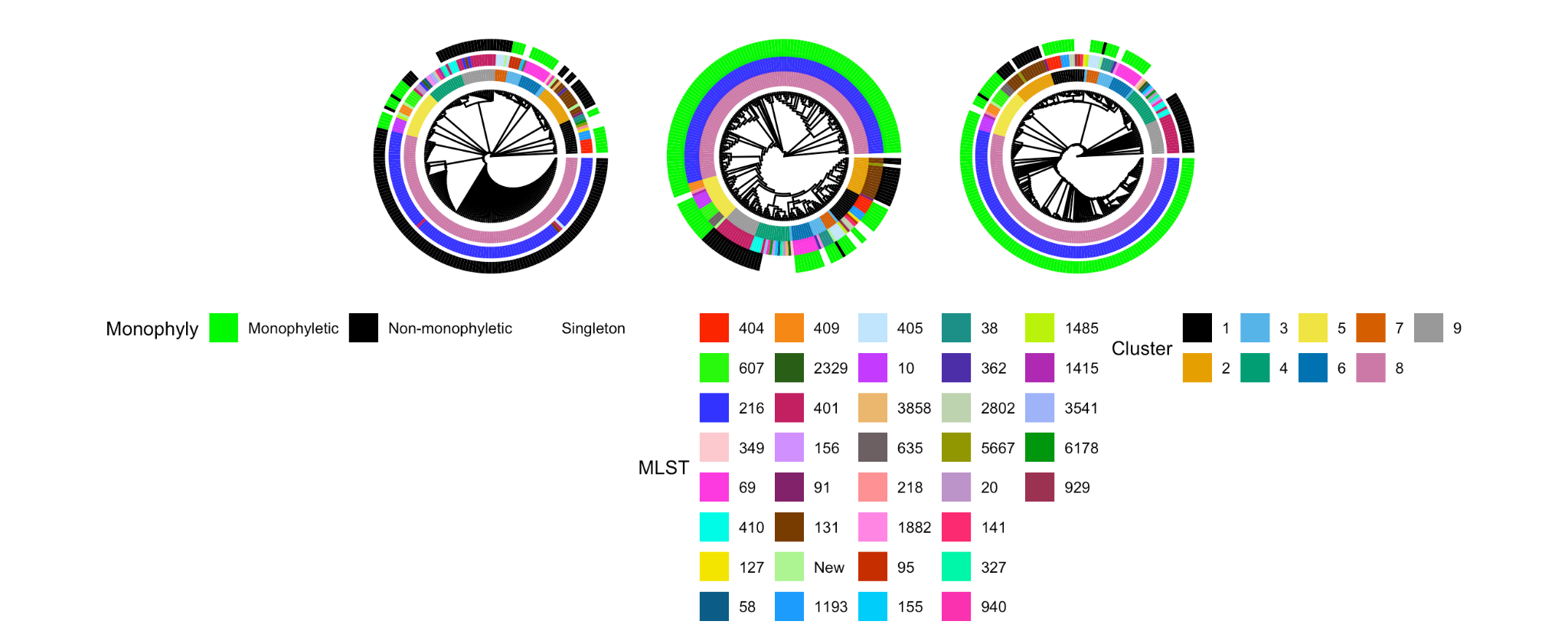


Supplementary Figure 11: Circular genetic trees assembled for the *long* outbreak including: (A) neighbour typing predicted genetic tree using neighbor typing; (B) reference genetic tree created using *mashtree*; (C) reference ML phylogeny created using *PanACoTA*. The outer ring denotes the MLST of each sample. Tips are coloured by cluster, as determined using *rhierBAPS*, and the clusters from the ML reference method are mapped onto the best match trees for comparison. An arbitrary sample number is labelled at the tips for ease of comparison of sample locations between trees.

[Alt text: Graphical representation of two genetic trees and one phylogeny for the *short* outbreak dataset, with subfigures labelled A-C. This is an updated version of Figure 4, with an additional heatmap layer describing MLST of the sample. Samples generally cluster by MLST, regardless of tree.]


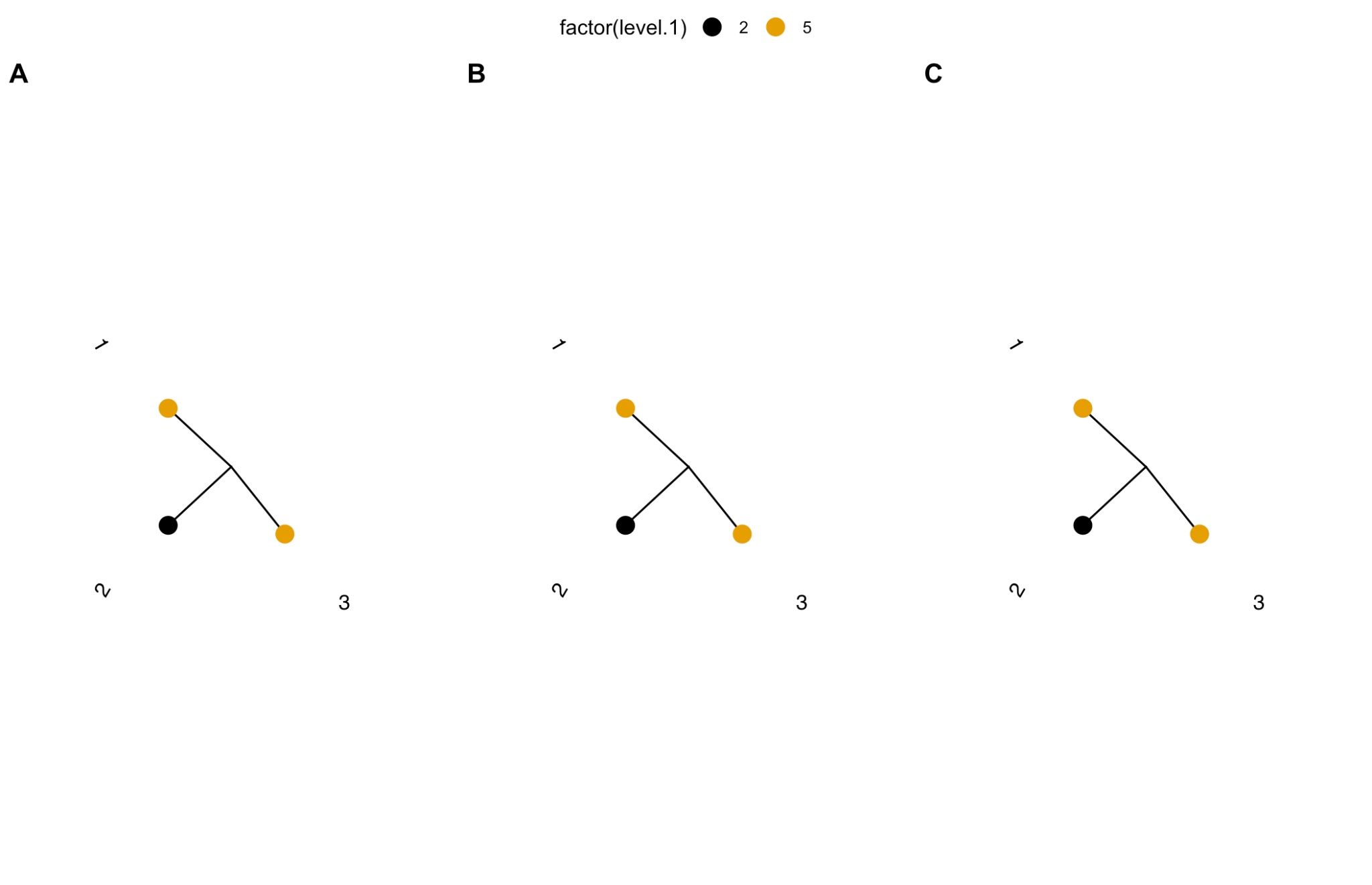


Supplementary Figure 12: Circular genetic trees assembled for the *long* outbreak for isolates from 2010 queried against the original database, including: (A) neighbour typing predicted genetic tree using neighbor typing; (B) reference genetic tree created using *mashtree*; (C) reference ML phylogeny created using *PanACoTA*. Tips are coloured by cluster, as determined using *rhierBAPS*, and the clusters from the ML reference method are mapped onto the best match trees for comparison. An arbitrary sample number is labelled at the tips for ease of comparison of sample locations between trees.

[Alt text: Graphical representation of two genetic trees and one phylogeny for the isolates collected in 2010 from the *long* outbreak dataset, with subfigures labelled A-C. There are two distinct coloured clusters observed in all subfigures, and all trees are clustering identically.]


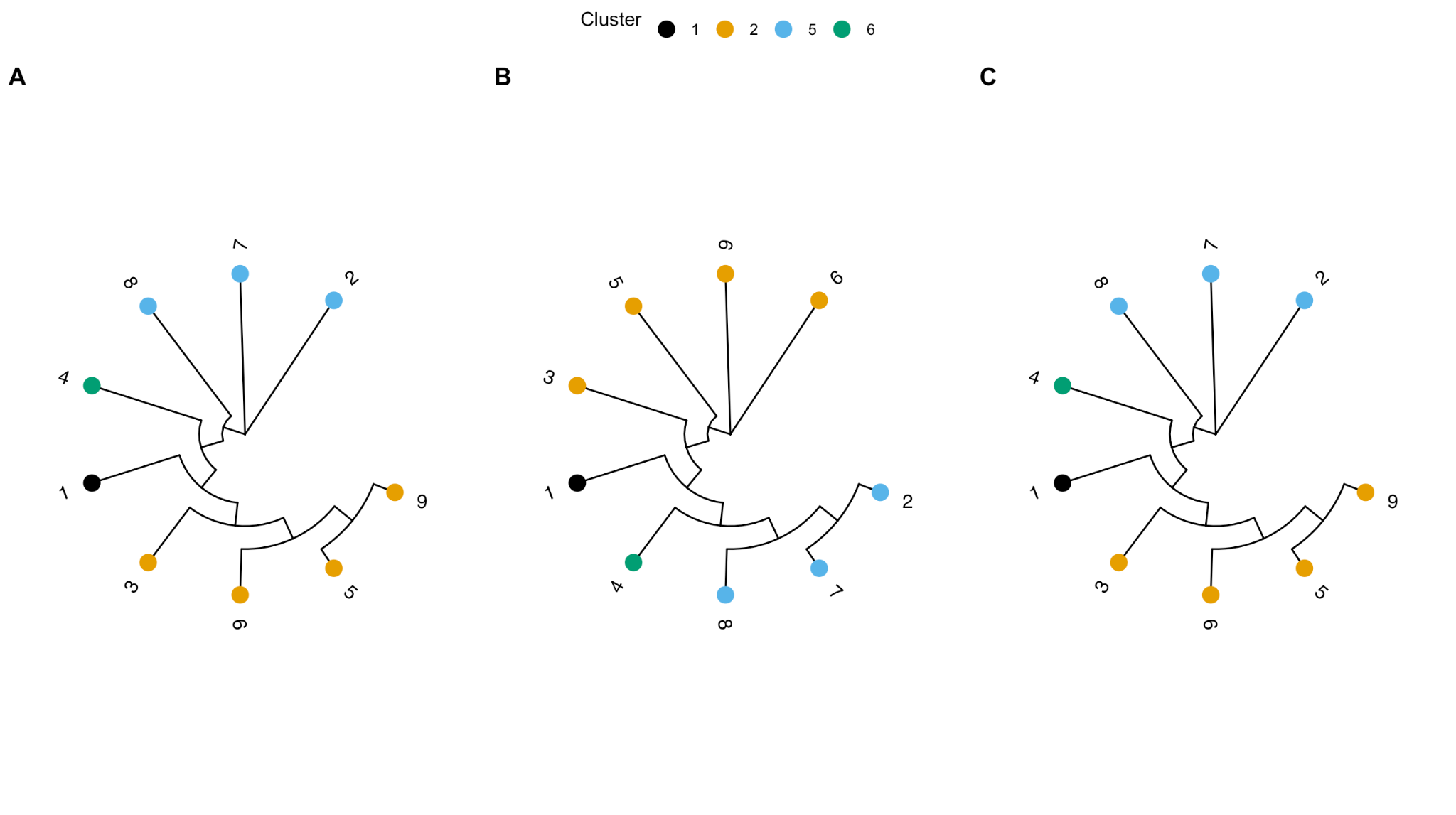


Supplementary Figure 13: Circular genetic trees assembled for the *long* outbreak for isolates from 2010-2011 queried against the original database supplemented with isolates from 2010, including: (A) neighbour typing predicted genetic tree using neighbor typing; (B) reference genetic tree created using *mashtree;* (C) reference ML phylogeny created using *PanACoTA*. Tips are coloured by cluster, as determined using *rhierBAPS*, and the clusters from the ML reference method are mapped onto the best match trees for comparison. An arbitrary sample number is labelled at the tips for ease of comparison of sample locations between trees.

[Alt text: Graphical representation of two genetic trees and one phylogeny for the isolates collected in 2010 and 2011 from the *long* outbreak dataset, with subfigures labelled A-C. There are four distinct coloured clusters observed in all subfigures, and all trees are clustering similarly.]


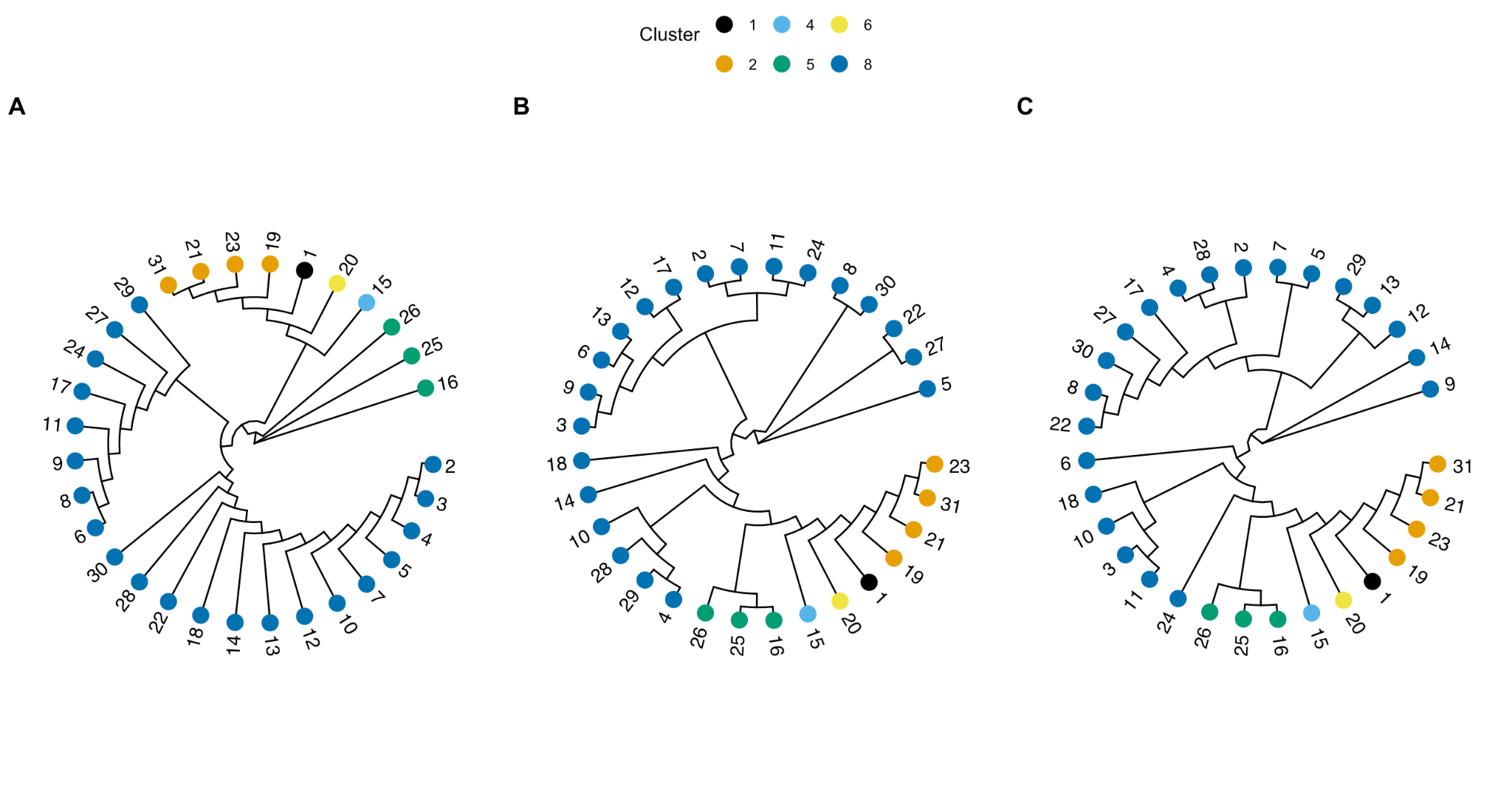


Supplementary Figure 14: Circular genetic trees assembled for the *long* outbreak for isolates from 2010-2012 queried against the original database supplemented with isolates up to 2011, including: (A) neighbour typing predicted genetic tree using neighbor typing; (B) reference genetic tree created using *mashtree*; (C) reference ML phylogeny created using *PanACoTA*. Tips are coloured by cluster, as determined using *rhierBAPS*, and the clusters from the ML reference method are mapped onto the best match trees for comparison. An arbitrary sample number is labelled at the tips for ease of comparison of sample locations between trees.

[Alt text: Graphical representation of two genetic trees and one phylogeny for the isolates collected from 2010-2012 from the long outbreak dataset, with subfigures labelled A-C. There are six distinct coloured clusters observed in all subfigures, and all trees are clustering similarly.]


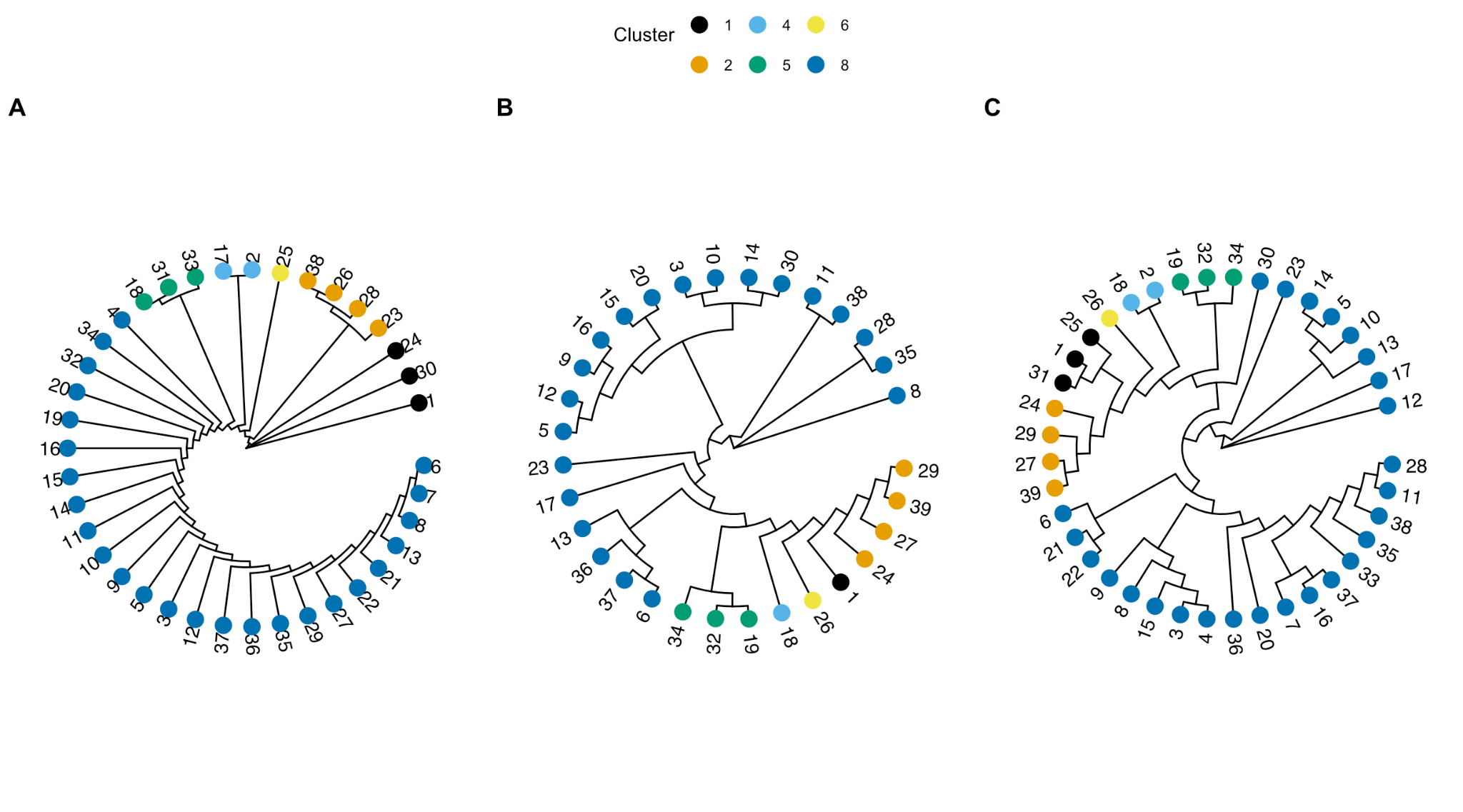


Supplementary Figure 15: Genetic trees assembled for the *long* outbreak for isolates from 2010-2013 queried against the original database supplemented with isolates up to 2012, including: (A) neighbour typing predicted genetic tree using neighbor typing; (B) reference genetic tree created using *mashtree*; (C) reference ML phylogeny created using *PanACoTA*. Tips are coloured by cluster, as determined using *rhierBAPS*, and the clusters from the ML reference method are mapped onto the best match trees for comparison. An arbitrary sample number is labelled at the tips for ease of comparison of sample locations between trees.

[Alt text: Graphical representation of two genetic trees and one phylogeny for the isolates collected from 2010-2013 from the long outbreak dataset, with subfigures labelled A-C. There are six distinct coloured clusters observed in all subfigures, and all trees are clustering similarly.]


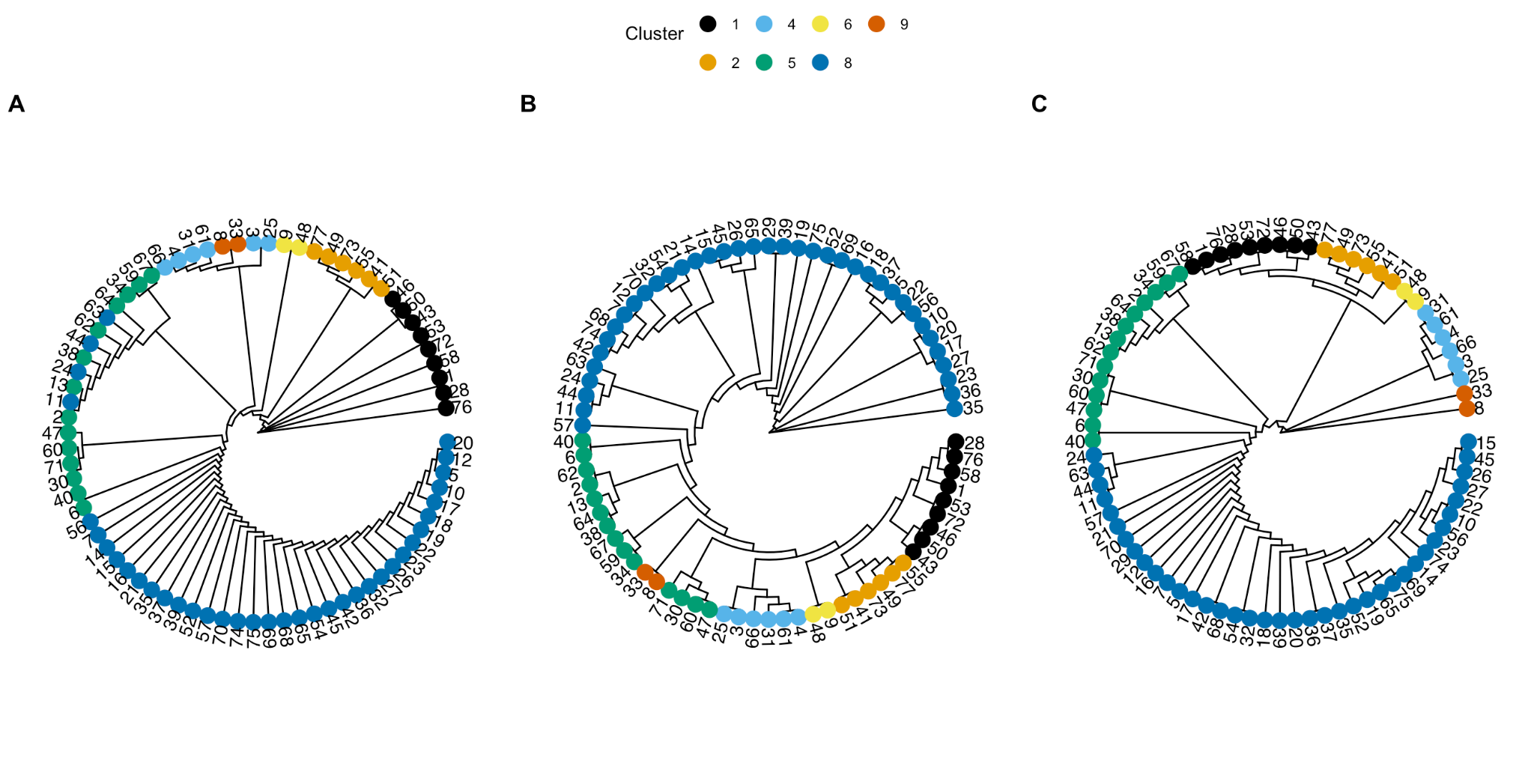


Supplementary Figure 16: Circular genetic trees assembled for the *long* outbreak for isolates from 2010-2014 queried against the original database supplemented with isolates up to 2013, including: (A) neighbour typing predicted genetic tree using neighbor typing; (B) Reference genetic tree created using *mashtree*; (C) Reference ML phylogeny created using *PanACoTA*. Tips are coloured by cluster, as determined using *rhierBAPS*, and the clusters from the ML reference method are mapped onto the best match trees for comparison. An arbitrary sample number is labelled at the tips for ease of comparison of sample locations between trees.

[Alt text: Graphical representation of two genetic trees and one phylogeny for the isolates collected from 2010-2014 from the long outbreak dataset, with subfigures labelled A-C. There are seven distinct coloured clusters observed in all subfigures, and all trees are clustering similarly.]


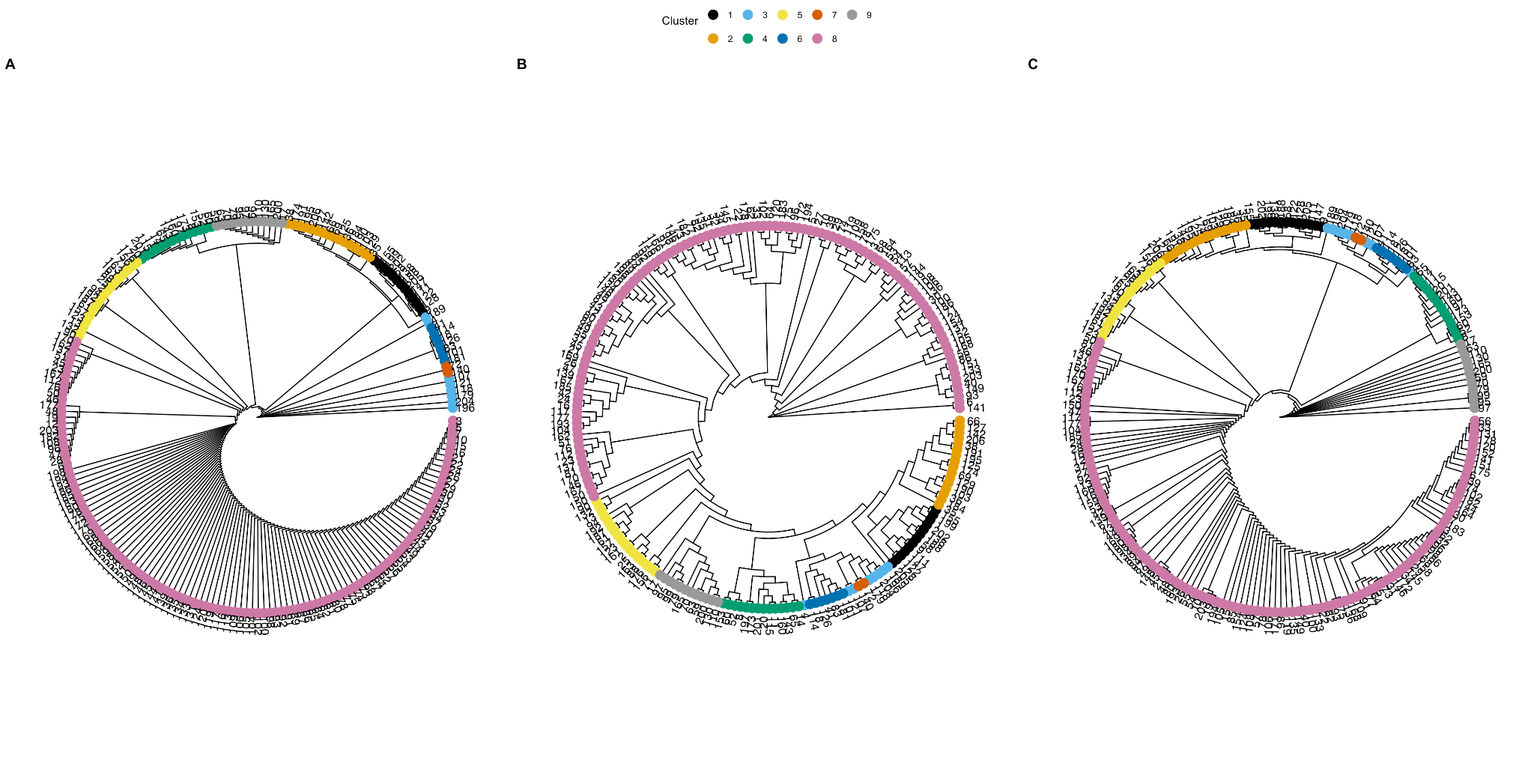


Supplementary Figure 17: Circular genetic trees assembled for the *long* outbreak for isolates from 2010-2015 queried against the original database supplemented with isolates up to 2014, including: (A) neighbour typing predicted genetic tree using neighbor typing; (B) reference genetic tree created using *mashtree*; (C) reference ML phylogeny created using *PanACoTA*. Tips are coloured by cluster, as determined using *rhierBAPS*, and the clusters from the ML reference method are mapped onto the best match trees for comparison. An arbitrary sample number is labelled at the tips for ease of comparison of sample locations between trees.

[Alt text: Graphical representation of two genetic trees and one phylogeny for the isolates collected from 2010-2015 from the long outbreak dataset, with subfigures labelled A-C. There are nine distinct coloured clusters observed in all subfigures, and all trees are clustering similarly.]

***Supplemental Tables***

Supplementary Table 1: MLSTs represented in the initial enhanced database used for initial neighbour typing, and the respective number of occurrences of each within the database.

| MLST | Occurrences (n) | Percentage (% of 202) |
| --- | --- | --- |
| 131 | 25 | 12.4 |
| 73 | 16 | 7.9 |
| 95 | 16 | 7.9 |
| 1193 | 12 | 5.9 |
| 10 | 9 | 4.5 |
| 69 | 9 | 4.5 |
| 12 | 6 | 3 |
| 38 | 5 | 2.5 |
| 127 | 5 | 2.5 |
| 404 | 4 | 2 |
| 58 | 3 | 1.5 |
| 141 | 3 | 1.5 |
| 144 | 3 | 1.5 |
| 349 | 3 | 1.5 |
| 357 | 3 | 1.5 |
| 1163 | 3 | 1.5 |
| 70 | 2 | 1 |
| 155 | 2 | 1 |
| 14 | 1 | 0.5 |
| 23 | 1 | 0.5 |
| 44 | 1 | 0.5 |
| 46 | 1 | 0.5 |
| 48 | 1 | 0.5 |
| 57 | 1 | 0.5 |
| 59 | 1 | 0.5 |
| 62 | 1 | 0.5 |
| 68 | 1 | 0.5 |
| 80 | 1 | 0.5 |
| 88 | 1 | 0.5 |
| 91 | 1 | 0.5 |
| 93 | 1 | 0.5 |
| 101 | 1 | 0.5 |
| 117 | 1 | 0.5 |
| 156 | 1 | 0.5 |
| 162 | 1 | 0.5 |
| 167 | 1 | 0.5 |
| 193 | 1 | 0.5 |
| 196 | 1 | 0.5 |
| 224 | 1 | 0.5 |
| 297 | 1 | 0.5 |
| 354 | 1 | 0.5 |
| 359 | 1 | 0.5 |
| 361 | 1 | 0.5 |
| 372 | 1 | 0.5 |
| 393 | 1 | 0.5 |
| 394 | 1 | 0.5 |
| 399 | 1 | 0.5 |
| 405 | 1 | 0.5 |
| 410 | 1 | 0.5 |
| 420 | 1 | 0.5 |
| 421 | 1 | 0.5 |
| 428 | 1 | 0.5 |
| 429 | 1 | 0.5 |
| 448 | 1 | 0.5 |
| 453 | 1 | 0.5 |
| 457 | 1 | 0.5 |
| 538 | 1 | 0.5 |
| 543 | 1 | 0.5 |
| 550 | 1 | 0.5 |
| 569 | 1 | 0.5 |
| 617 | 1 | 0.5 |
| 624 | 1 | 0.5 |
| 636 | 1 | 0.5 |
| 646 | 1 | 0.5 |
| 648 | 1 | 0.5 |
| 655 | 1 | 0.5 |
| 744 | 1 | 0.5 |
| 746 | 1 | 0.5 |
| 964 | 1 | 0.5 |
| 973 | 1 | 0.5 |
| 998 | 1 | 0.5 |
| 1161 | 1 | 0.5 |
| 1177 | 1 | 0.5 |
| 1332 | 1 | 0.5 |
| 1431 | 1 | 0.5 |
| 1722 | 1 | 0.5 |
| 2003 | 1 | 0.5 |
| 2526 | 1 | 0.5 |
| 2556 | 1 | 0.5 |
| 2562 | 1 | 0.5 |
| 2617 | 1 | 0.5 |
| 3580 | 1 | 0.5 |
| 4358 | 1 | 0.5 |
| 4448 | 1 | 0.5 |
| 4623 | 1 | 0.5 |
| 6823 | 1 | 0.5 |
| 9075 | 1 | 0.5 |
| 9505 | 1 | 0.5 |
| 9535 | 1 | 0.5 |
| 12159 | 1 | 0.5 |
| 12261 | 1 | 0.5 |

Table S2: Summary of database contents and samples queried in each iteration for the *long* outbreak analysis [(Decraene et al. 2018)](https://paperpile.com/c/a2agwD/jmNt8).

| Database Contents | Samples Queried |
| --- | --- |
| Original database [(Carroll et al. 2024)](https://paperpile.com/c/a2agwD/Lsrdi) | 2010 |
| Original with 2010 isolates | 2010 and 2011 |
| Original with 2010, 2011 isolates | 2010, 2011, 2012 |
| Original with 2010, 2011, 2012 isolates | 2010, 2011, 2012, 2013 |
| Original with 2010, 2011, 2012, 2013 isolates | 2010, 2011, 2012, 2013, 2014 |
| Original with 2010, 2011, 2012, 2013, 2014 isolates | 2010, 2011, 2012, 2013, 2014, and 2015 |
| Original with 2010, 2011, 2012, 2013, 2014, 2015 isolates | 2010, 2011, 2012, 2013, 2014, 2015, and 2016 |
